# Identification of novel and salt-responsive miRNAs to explore miRNA-mediated regulatory network of salt stress response in radish (*Raphanus sativus* L.)

**DOI:** 10.1186/s12864-015-1416-5

**Published:** 2015-03-17

**Authors:** Xiaochuan Sun, Liang Xu, Yan Wang, Rugang Yu, Xianwen Zhu, Xiaobo Luo, Yiqin Gong, Ronghua Wang, Cecilia Limera, Keyun Zhang, Liwang Liu

**Affiliations:** National Key Laboratory of Crop Genetics and Germplasm Enhancement, College of Horticulture, Nanjing Agricultural University, Nanjing, 210095 P.R. China; Jiangsu Key Laboratory for Horticultural Crop Genetic Improvement, Nanjing, 210014 P.R. China; Department of Plant Sciences, North Dakota State University, Fargo, ND 58108 USA; College of Life Sciences, Nanjing Agricultural University, Nanjing, 210095 P.R.China

**Keywords:** Radish (*Raphanus sativus* L*.*), Salt stress, MicroRNA, Target gene, RT-qPCR, High-throughput sequencing

## Abstract

**Background:**

Salt stress is one of the most representative abiotic stresses that severely affect plant growth and development. MicroRNAs (miRNAs) are well known for their significant involvement in plant responses to abiotic stresses. Although miRNAs implicated in salt stress response have been widely reported in numerous plant species, their regulatory roles in the adaptive response to salt stress in radish (*Raphanus sativus* L.), an important root vegetable crop worldwide, remain largely unknown.

**Results:**

Solexa sequencing of two sRNA libraries from NaCl-free (CK) and NaCl-treated (Na200) radish roots were performed for systematical identification of salt-responsive miRNAs and their expression profiling in radish. Totally, 136 known miRNAs (representing 43 miRNA families) and 68 potential novel miRNAs (belonging to 51 miRNA families) were identified. Of these miRNAs, 49 known and 22 novel miRNAs were differentially expressed under salt stress. Target prediction and annotation indicated that these miRNAs exerted a role by regulating specific stress-responsive genes, such as squamosa promoter binding-like proteins (*SPLs*), auxin response factors (*ARFs*), nuclear transcription factor Y (*NF-Y*) and superoxide dismutase [Cu-Zn] (*CSD1*). Further functional analysis suggested that these target genes were mainly implicated in signal perception and transduction, regulation of ion homeostasis, basic metabolic processes, secondary stress responses, as well as modulation of attenuated plant growth and development under salt stress. Additionally, the expression patterns of ten miRNAs and five corresponding target genes were validated by reverse-transcription quantitative PCR (RT-qPCR).

**Conclusions:**

With the sRNA sequencing, salt-responsive miRNAs and their target genes in radish were comprehensively identified. The results provide novel insight into complex miRNA-mediated regulatory network of salt stress response in radish, and facilitate further dissection of molecular mechanism underlying plant adaptive response to salt stress in root vegetable crops.

**Electronic supplementary material:**

The online version of this article (doi:10.1186/s12864-015-1416-5) contains supplementary material, which is available to authorized users.

## Background

Salt stress is one of the major environmental threats that negatively affect plant growth and development. Approximately 20% of agricultural land and 50% of cropland worldwide are subjected to salt exposure [1]. Irrigation water containing trace amounts of sodium chloride (NaCl) and seawater is main source of salt in soil [2]. Increasing soil salinity lowers the ability of plant to take up water, and excess ions like Na^+^ and Cl^−^ absorbed by roots are harmful to growth of plant by injuring metabolic processes and decreasing photosynthetic efficiency [3]. High salinity also imposes secondary stresses like nutritional imbalance and oxidative stress that result in cell damage, yield decrease and growth inhibition. To address these challenges, genetically engineering plants to enhance salt tolerance will be a promising approach. Therefore, deciphering the physiological processes and molecular genetic mechanism related to salt-stress resistance will certainly facilitate the understanding of complex biological responses of plants against high salinity, and help in genetically engineering of stress-resistant plants.

MicroRNAs (miRNAs) are a series of endogenous small non-coding RNA molecules, which negatively regulate gene expression at transcriptional and post-transcriptional levels by modulating both mRNA degradation and translational suppression based on sequence complementarity with their target(s) [4]. In plants, a long primary transcript known as miRNA primary precursor (pri-miRNA) is transcribed from a nuclear-encoded miRNA gene. Then, a miRNA:miRNA* duplex is released from the fold-back stem loop of miRNA precursor obtained from pri-miRNA by two cuts under the guide of Dicer-like 1 (DCL1) assisted by the dsRNA binding protein HYL1 [5]. Finally, the mature miRNA is methylated by HEN1 [6], and then bound with the argonaute protein 1 (AGO1) to form a functional special structure named RNA-induced silencing complex (RISC), which targets specific mRNAs and suppresses their expression by cleavage. Aside from the roles in modulating a wide range of essential biochemical, molecular and physiological processes, many studies reported that miRNAs were involved in plant responses to a variety of abiotic stresses such as salt [7,8], drought [9,10], heat [11,12], cold [7,13] and oxidative stress [14].

Understanding the miRNA-mediated regulatory network of salt stress response will lay the foundation for unraveling the complex molecular genetic mechanism of salt-stress tolerance. A growing body of evidences suggested that miRNA-guided gene regulation could play a vital role in plant response to salt stress. Using microarray approach, several miRNAs such as miR156, miR159, miR167, miR168, miR171, miR319 and miR396 were found to be differentially expressed after salt shock in *Arabidopsis* [7] and *Zea mays* [15]. Recently, the extensive application of next generation sequencing (NGS) technology has provided unparalleled opportunities to obtain comprehensive sequencing data for the detection of salt-responsive miRNAs in various plant species. Using this approach, Dong et al. [16] identified 104 differentially expressed miRNAs in salt-stressed functional soybean nodules. Under salt stress, seven downregulated conserved miRNA families and two upregulated miRNA families were isolated in *Populus tomentosa* [8]*.* Also in *Populus euphratica*, 132 miRNAs showed expression alterations during salt stress [17]. In salt-stressed *Caragana intermedia*, the expression of seven miRNAs including cin-miR157a, cin-miR159a, cin-miR165a, cin-miR167b, cin-miR172b, cin-miR390a and cin-miR396a were induced, while cin-miR398a was repressed [18]. Additionally, many salt-responsive miRNAs were also been identified in some vegetable crops. For instance, in soybeans, 50 miRNAs were detected to be differentially expressed under salt treatment [19]. Differentially expressed miRNAs including 11 downregulated and three upregulated ones were identified after salt stress in *Solanum linnaeanum* using NGS technology [20]. Moreover, 11 miRNAs were identified to be differentially regulated by abiotic stresses (heat, cold, salinity and drought) in celery [21]. Totally, 42 known and 39 candidate miRNAs were differentially expressed under salt condition in broccoli [22]. Taken together, these findings implied that miRNA-mediated gene regulatory pathways could play significant roles in plant adaptive response towards salt stress.

Radish (*Raphanus sativus* L., 2n = 18), belonging to the Brassicaceae family, is a globally important root vegetable crop especially in Asia [23]. Using Solexa-sequencing technology, Xu et al. [24] identified 545 conserved miRNA families and 79 novel miRNAs from radish roots. More recently, some conserved and novel miRNAs associated with cadmium stress response, embryogenesis and lead stress response were also identified in radish [25-27]. Salt stress is a limiting factor for radish that adversely influences germination, fresh weight, health-promoting compounds and antioxidant activity [28]. Therefore, exploring the regulatory mechanism responsive to salt stress will be of important significance for engineering of salt-tolerant radish germplasm. However, no investigation on identification of miRNAs and their target genes responsive to salt stress in radish has been reported to date. In this study, two small RNA (sRNA) libraries from the control (NaCl-free) and salt-treated (200 mM NaCl for 48 h) radish roots were constructed and sequenced using NGS technology. The aims were to detect salt-responsive miRNAs from radish roots, explore their roles in plant response to salt stress by predicting their target transcripts, and reveal the miRNA-mediated regulatory network of salt stress response in radish. The obtained results of this study could provide valuable information for further validating the regulatory roles of salt-responsive miRNAs in radish, and facilitate dissection of molecular mechanism underlying plant adaptive response to salt stress in radish and other root vegetable crops.

## Results

### Overview of transcriptome and sRNA sequencing in radish

To establish an overall reference sequence database, a cDNA library constructed from radish roots was sequenced, totally 57.03 M raw reads were generated and 130,953 contigs were obtained [NCBI Sequence Read Archive (SRA) with the GenBank accession No.SRS706782]. These mRNA transcriptome sequences, together with the available GSS (Genome Survey Sequence) and EST (Expressed Sequence Tag) sequences released in NCBI database, formed the radish reference genome for identification of known and novel miRNAs in radish, as well as the prediction of miRNA corresponding target genes.

Radish seedlings under salt treatment exhibited some negatively morphological changes including chlorisis and withering of leaves, and slight inhibition of plant growth. In this study, 18.38 M and 17.49 M raw reads were generated from CK and Na200 libraries, respectively (Table 1). After filtering out adapter contamination and low-quality tags, 18.13 M (representing 3,370,688 unique sequences) and 17.24 M (representing 4,200,793 unique sequences) clean reads were acquired from CK and Na200 libraries, respectively (Tables 1 and 2). Among them, 3,947,380 (11.16%) were CK library-specific with 2,565,929 (37.92%) unique sequences, 4,563,071 (12.90%) were only derived from Na200 library with 3,396,034 (50.19%) unique sRNAs, and 26,861,034 (75.94%) were shared in both with 804,759 (11.89%) unique sequences (Table 3).

**Table 1:** **Summary of cleaning data from CK and Na200 sRNA libraries of radish roots**

**Type**	**CK**		**Na200**	
	**Count**	**Percent (%)**	**Count**	**Percent (%)**
Total_reads	18,381,536		17,486,008	
High_quality	18,301,525	100	17,412,468	100
3′adapter_null	2,335	0.01	2,221	0.01
Insert_null	1,600	0.01	4,224	0.02
5′adapter_contaminants	94,403	0.52	81,947	0.47
Smaller_than_18nt	72,800	0.4	79,371	0.46
Poly(A)	1,230	0.01	2,377	0.01
clean_reads	18,129,157	99.06	17,242,328	99.02

**Table 2:** **Distribution of small RNAs among different categories in radish**

**Category**	**CK**		**Na200**	
	**Unique sRNAs**	**Total sRNAs**	**Unique sRNAs**	**Total sRNAs**
Total	3,370,688(100%)	18,129,157(100%)	4,200,793(100%)	17,242,328(100%)
miRNA	18,165(0.54%)	1,619,066(8.93%)	19,429(0.46%)	3,221,269(18.68%)
rRNA	145,346(4.31%)	2,251,398(12.42%)	204,170(4.86%)	3,442,702(19.97%)
snRNA	4,403(0.13%)	15,898(0.09%)	6,650(0.16%)	27,950(0.16%)
snoRNA	2,292(0.07%)	4,553(0.03%)	2,976(0.07%)	6,963(0.04%)
tRNA	10,292(0.31%)	260,694(1.44%)	20,295(0.48%)	295,998(1.72%)
Unannotated	3,190,190(94.65%)	13,977,548(77.10%)	3,947,273(93.96%)	10,247,446(59.43%)

**Table 3:** **Summary of common and specific sequences between CK and Na200 sRNA libraries**

**Class**	**Unique sRNAs**	**Percentage**	**Total sRNAs**	**Percentage**
Total_sRNAs	6,766,722	100.00%	35,371,485	100.00%
NaCl_200&CK	804,759	11.89%	26,861,034	75.94%
NaCl_200_specific	3,396,034	50.19%	4,563,071	12.90%
CK_specific	2,565,929	37.92%	3,947,380	11.16%

**Figure 1:**
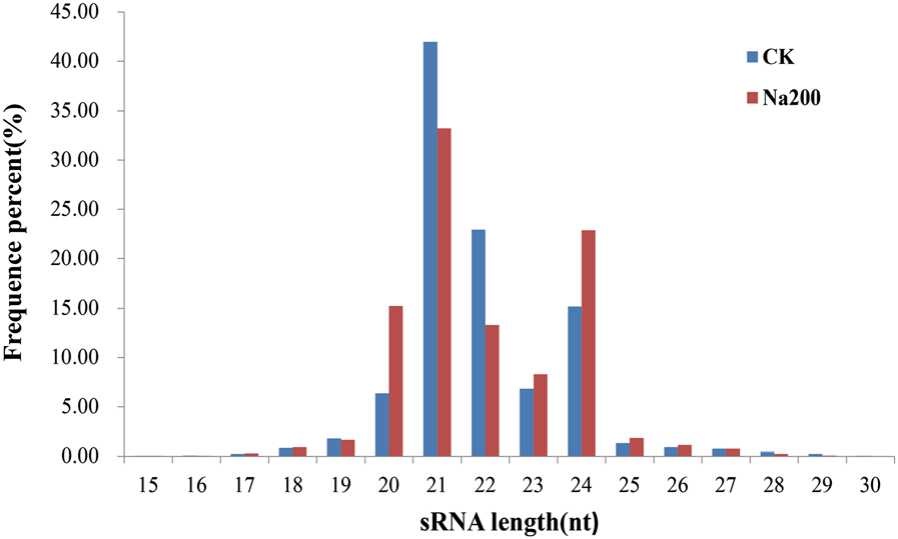
**Length distribution of small RNAs in CK and Na200 libraries from radish roots.** Y-axis represents percentages of sRNAs identified in this study; X-axis represents the length of sRNAs. Two libraries are shown by different colors.

The sRNA size distribution in both libraries was summarized in Figure 1. The most abundant sRNAs ranged from 20 to 24 nt, and the 21 nt sRNAs represented the most frequent length (41.96% in CK and 33.22% in Na200 library, respectively), which was in agreement with previous reports in other plant species including trifoliate orange [13], *Populus* [8,29] and grapevine [30]. Furthermore, these sRNAs were annotated into several different categories (Table 2). Of these, 18,165 (0.54%) and 19,429 (0.46%) unique sRNAs were annotated as miRNAs in CK and Na200 libraries, respectively. Additionally, a predominant proportion of unique sequences (> 90% in two libraries) were unannotated sRNAs, suggesting a broad existence of novel miRNAs in radish.

### Identification of known miRNAs in radish

**Figure 2:**
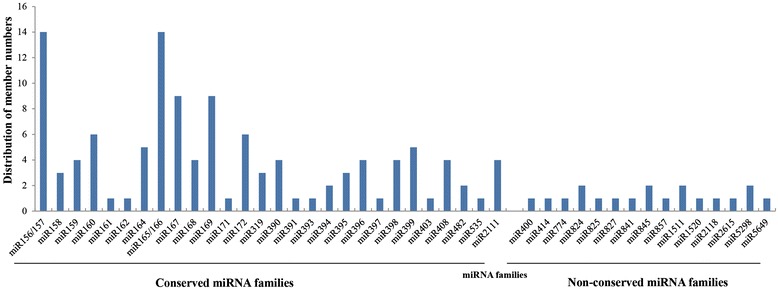
**Distribution of known miRNA family size in** ***R. sativus.*** Y-axis represents various known miRNA families identified in this study. X-axis represents the number of members for each miRNA family.

A total of 117 unique sequences belonging to 28 conserved miRNA families and 19 unique sequences representing 15 non-conserved miRNA families were identified in both libraries (Table 4 and Additional file 1). The diversity of radish miRNA families could be determined from their number of members (Figure 2). As shown, most of the conserved miRNA families had members of no less than two. Of these, the miR156/157 and miR165/166 families were the largest ones with 14 members, followed by miR167 and miR169 with nine members. However, some conserved miRNA families including miR161, miR162, miR171, miR391, miR393, miR397, miR403 and miR535, had only one member. Moreover, most of the non-conserved miRNA families contained only one member.

**Table 4:** **Known miRNA families and their expression abundance in CK and Na200 libraries**

**Family**	**No. of members**	**miRNA reads**		**Total reads**	**Ratio (Na200/CK)**
		**CK**	**Na200**		
**Conserved miRNA**					
miR156/157	14	390,906	477,699	868,605	1.22
miR158	3	699,743	3,592,759	4,292,502	5.13
miR159	4	3,509	2,881	6,390	0.82
miR160	6	4,119	3,776	7,895	0.92
miR161	1	0	10	10	_
miR162	1	750	922	1,672	1.23
miR164	5	21,869	70,350	92,219	3.22
miR165/166	14	91,496	208,941	300,437	2.28
miR167	9	24,223	19,501	43,724	0.81
miR168	4	196,762	319,663	516,425	1.62
miR169	9	10,148	2,552	12,700	0.25
miR171	1	155	28	183	0.18
miR172	6	1,486	4,762	6,248	3.20
miR319	3	14,416	17,633	32,049	1.22
miR390	4	431	911	1,342	2.10
miR391	1	1,066	1,993	3,059	1.87
miR393	1	15	13	28	0.87
miR394	2	201	416	617	2.07
miR395	3	95	48,134	48,229	506.67
miR396	4	981	1,177	2,158	1.20
miR397	1	15,201	6,493	21,694	0.43
miR398	4	2,032	326	2,358	0.16
miR399	5	249	150	399	0.60
miR403	1	1,634	2,649	4,283	1.62
miR408	4	269,431	115,871	385,302	0.43
miR482	2	145	1,543	1,688	10.64
MiR535	1	0	1,524	1,524	_
miR2111	4	341	122	463	0.36
**Non-conserved miRNA**					
miR400	1	137	151	288	1.10
miR414	1	344	0	344	0.00
miR774	1	3,704	4,062	7,766	1.10
miR824	2	529	619	1,148	1.17
miR825	1	1,455	1,861	3,316	1.28
miR827	1	275	230	505	0.84
miR841	1	0	293	293	_
miR845	2	3,003	7,471	10,474	2.49
miR857	1	1,413	819	2,232	0.58
miR1511	2	10,593	25,438	36,031	2.40
miR1520	1	17,011	17,004	34,015	1.00
miR2118	1	27,628	20,177	47,805	0.73
miR2615	1	7,581	1,074	8,655	0.14
miR5298	2	21	39	60	1.86
miR5649	1	35	45	80	1.29

**Figure 3:**
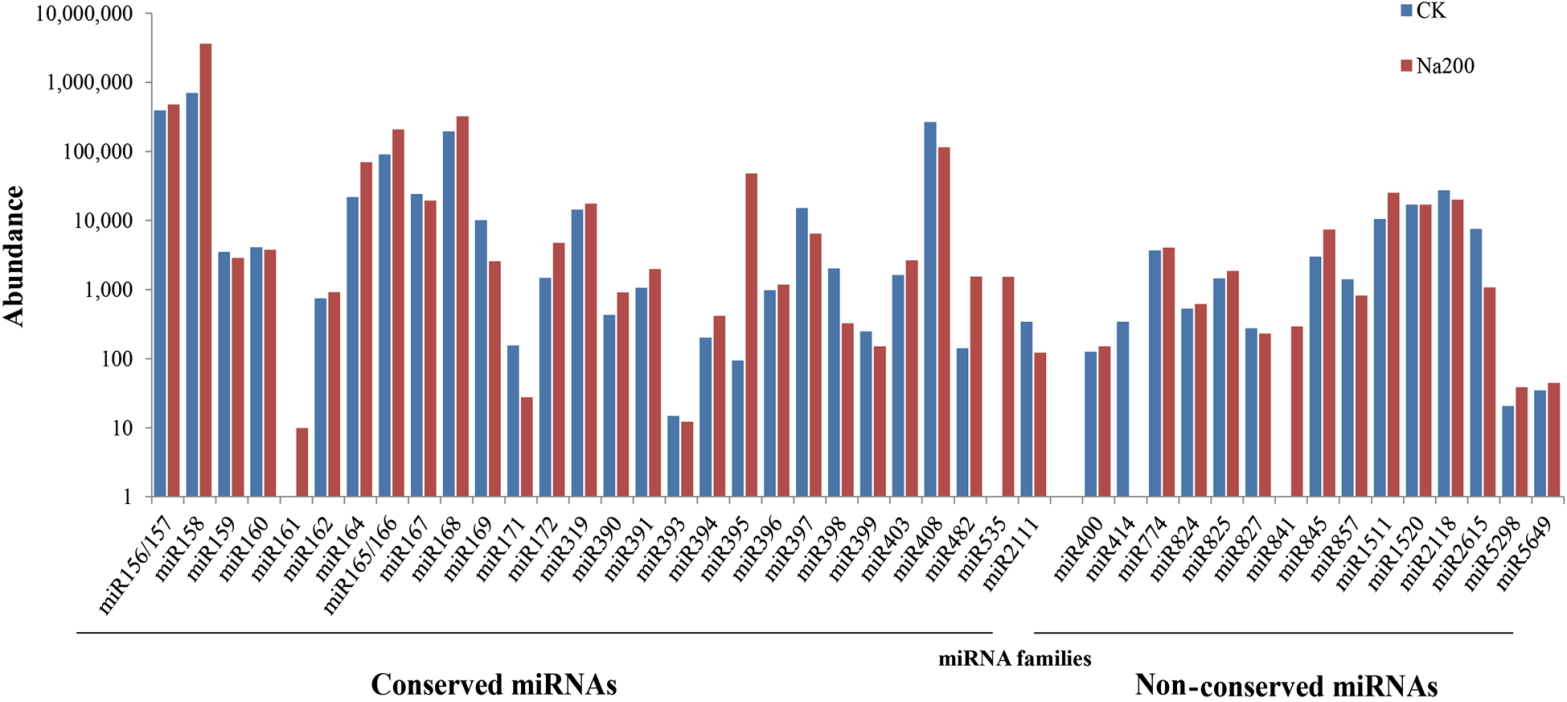
**Abundance of each known miRNA family in** ***R. sativus.*** Y-axis represents various known miRNA families and X-axis represents the abundance of each miRNA family in this study.

The number of miRNA reads in two libraries was highly variable with a ratio (Na200/CK) ranging from 0 to 506.67, and was exploited as the indicator for assessing miRNA expression level (Figure 3 and Table 4). miR158 presented the highest expression abundance with 699,743 and 3,592,759 copies in CK and Na200 libraries, respectively. Several conserved miRNA families including miR156/157, miR165/166, miR168 and miR408 also exhibited extraordinarily high abundance in both libraries, while some other miRNA families (miR164, miR167, miR169, miR319, miR395, miR397, miR845, miR1511, miR1520 and miR2118) were moderately expressed with a total reads ranging from 100,000 to 1,000,000. However, several miRNA families including miR161, miR393, miR5298 and miR5649 were detected to be expressed at an extremely low level in both libraries. Furthermore, a significant distinction in expression abundance was also observed among different members in a certain miRNA family (Additional file 1). For example, in the miR156/157 family, the read number of miR157a was 589,530, while miR156h had only 22 copies. This vast expression span among different members within a family suggested the precise expression of miRNAs under certain conditions.

### Identification of novel miRNA candidates

In the present study, based on the recent annotated criteria of novel miRNAs [31], 68 sRNA unique sequences belonging to 51 miRNA families were identified as putative novel miRNAs. Of these, seven novel miRNAs were detected to have miRNA*s (complementary miRNA sequences), and many miRNA*s were sequenced only once (Table 5 and Additional file 2), which might be partially attributed to that most of the miRNA*s had been degraded in miRNA pathway [13]. Among these novel miRNAs, most of them were CK-specific or Na200-specific, which accounted for 31.65% and 35.44%, respectively. In addition, the novel mature miRNAs displayed a main length distribution ranging from 21 nt to 24 nt, and 21 nt miRNAs accounted for the highest proportion (65.43%). Regarding their expression abundance, only 20 novel miRNAs (29.41%) had been sequenced more than 100 copies in each sRNA library. Moreover, the precursors of 68 potential novel miRNAs were also predicted, with minimum free energy (MFE) values ranging from −98.7 to −18.0 kcal/mol and an average length of 148 nt (Additional file 3).

**Table 5:** Novel miRNAs with their complementary miRNA*s under salt stress in radish

**miRNA**	**Sequence (5′–3′)**	**Length (nt)**	**Precursor length (nt)**	**Count**		**MFE (kcal/mol)**	**Arm**	**miRNA location**
				**CK**	**Na200**			
rsa-mir2-5p	AAAUCAUACUUUCAUUGAUA	20	185	277	0	−71.3	5′	CL10794.Contig2
rsa-mir2-3p	UCAAUGAAAGGUAUGAUUCCC	21	185	277	1,228	−71.3	3′	CL10794.Contig2
rsa-mir4-5p	ACGUUUCUCGAACUCAAGACC	21	107	0	3	−64.5	5′	FY453420
rsa-mir4-3p	UCUUGAGUUCGAGGGACGCCA	21	107	115	193	−64.5	3′	FY453420
rsa-mir11-5p	AGGCGAUGAUGGAUACCGAGAA	22	91	310	0	−31.8	5′	CL2420.Contig13
rsa-mir11-3p	CUCGGUAGCGAUGGUUCAAUCUCG	24	91	1	0	−31.8	3′	CL2420.Contig13
rsa-mir13-5p	AUAUACUGAAGUUUAUACUCU	21	208	33	57	−33.5	5′	EY928450
rsa-mir13-3p	AUCAUAAAAUCUUCAUUAUCUAG	23	208	1	1	−33.5	3′	EY928450
rsa-mir22-5p	UGGUGCAGGUCGGGAACUGAU	21	110	13	0	−57.7	5′	EY910368
rsa-mir22-3p	CGGAUCCCGCCUUGUAUCAAG	21	110	1	0	−57.7	3′	EY910368
rsa-mir24-5p	CGGUUAGCUUGGAAGCCAAAA	21	178	0	1	−41.2	5′	CL10961.Contig1
rsa-mir24-3p	UUGUUUUCUGAGAAAAUGGGC	21	178	0	10	−41.2	3′	CL10961.Contig1
rsa-mir35b-5p	UCGACGGGAAGGGGCUUUCUCU	22	72	0	1	−20	5′	CL7248.Contig2
rsa-mir35b-3p	GGAAUGUUGUUUGGCUCGAAG	21	72	94	64	−20	3′	CL7248.Contig2

MFE (kcal/mol), minimal folding free energy.

### Identification of salt-responsive miRNAs in radish

**Figure 4:**
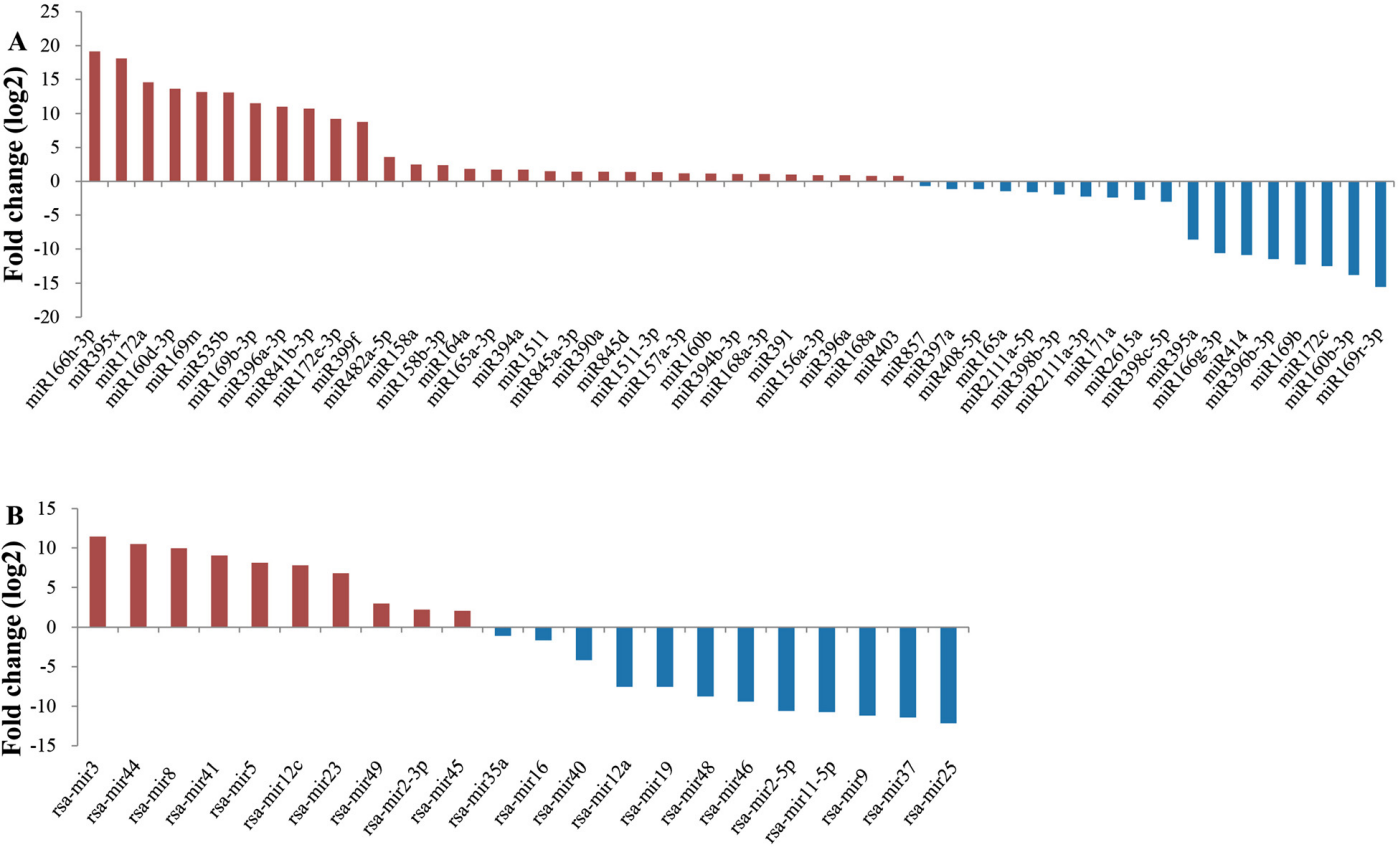
**Comparative relative expression of differentially expressed miRNAs.** Y-axis represents various differentially expressed miRNAs under salt stress in radish; X-axis represents the fold change value of each miRNA between Na200 and CK libraries. **A** represents differentially expressed known miRNAs and **B** represents differentially expressed novel miRNAs. The miRNAs with positive fold change value are upregulated, and the ones with negative fold change value are downregulated.

To identify the differentially expressed miRNAs under salt stress, the analysis of differential expression patterns was performed by statistical comparison between two libraries. In total, 49 known and 22 novel miRNAs were identified to be differentially regulated under salt stress (Additional file 4), and their expression alterations were showed in Figure 4. Among them, 41 miRNAs (31 known and 10 novel ones) were upregulated, and 30 miRNAs (18 known and 12 novel ones) were repressed by salt stress. Of these, 23 miRNAs including 16 known and seven novel miRNAs were markedly differentially expressed with an absolute value of log_2_ ratio (Na200/CK) > 10. In addition, many of these salt-responsive miRNAs, such as miR160b/d-3p, miR166g/h-3p, miR535b and rsa-mir3, were confined to be expressed in CK or Na200 library, suggesting that these miRNAs might be thoroughly induced or repressed by salt stress. Further analysis also indicated that different members in a certain miRNA family might have similar or disparate expression patterns after salt exposure. For example, miR172a and miR172e-3p were significantly upregulated, whereas miR172c was downregulated, providing further evidence on the complexity of miRNA regulatory roles.

### Prediction and annotation of target genes for salt-responsive miRNAs

**Figure 5:**
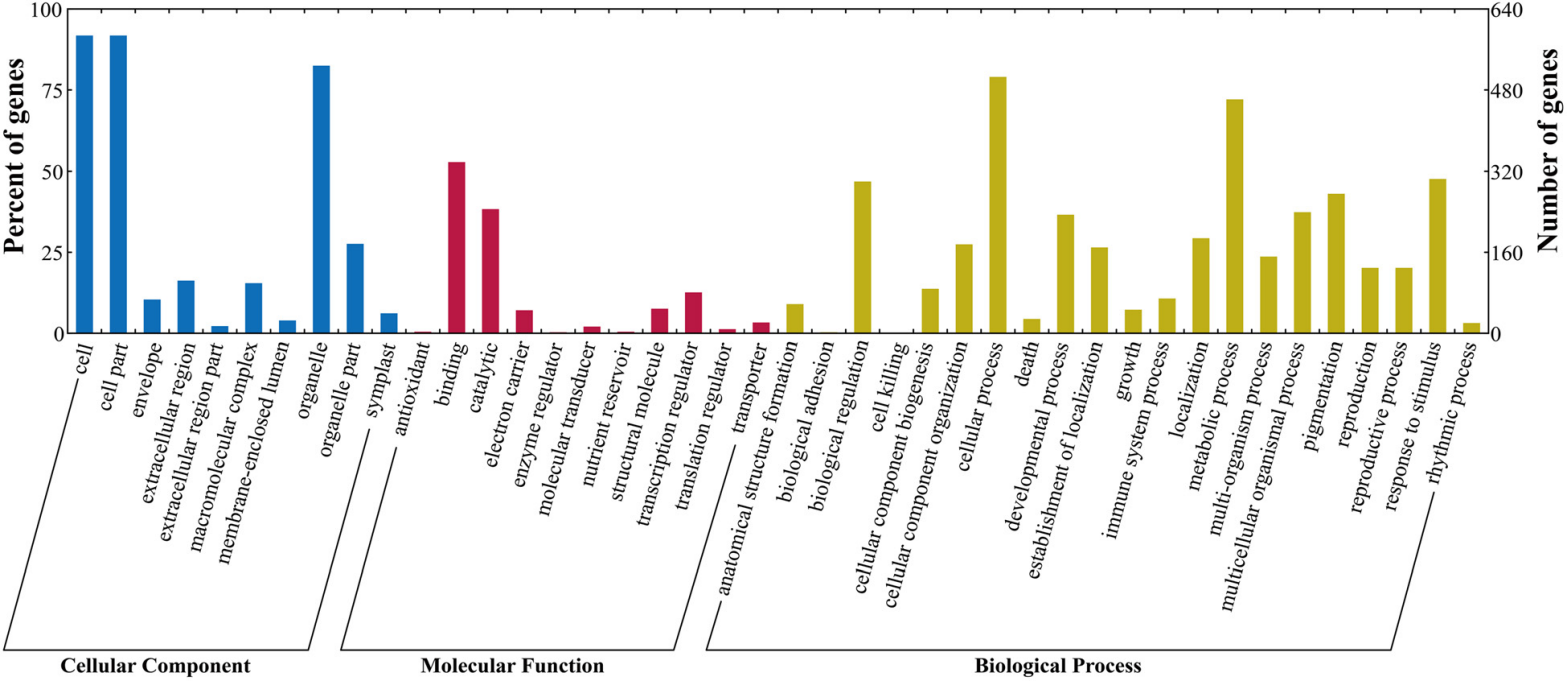
**Gene ontology classification of potential target genes for differentially expressed miRNAs.** Blue, red and yellow represent three GO ontologies: cellular component, molecular function and biological progress, respectively.

Predicting the target genes of miRNAs would be essential for better understanding of the biological functions for these salt-responsive miRNAs. As a result, 581 genes were predicted to be targets for 65 salt-responsive miRNAs (Table 6 and Additional file 5). All target sequences were successfully classified into three GO (Gene Ontology) ontologies using blast2go program, including cellular components, molecular functions and biological processes (Figure 5). As shown, the main terms were “cell” (GO: 0005623), “cell part” (GO: 0044464) and “organelle” (GO: 0043226) in the cellular component category. For their molecular functions, the “binding” (GO: 0005488) and “catalytic activity” (GO: 0003824) were the most abundant subcategories. The predominant terms implicated in biological processes were “biological regulation” (GO: 0065007), “cellular progress” (GO: 0009987), “developmental progress” (GO: 0032502), “metabolic progress” (GO: 0008152) and “response to stimulus” (GO: 0050896).

**Table 6:** **The target genes for some salt-responsive known miRNAs**

**miRNA**	**Target gene No.**	**Description**	**Gene name**
miR156/157	CL5609.Contig1	ethylene-responsive transcription factor ERF113	*Rap2.6 L*
	CL967.Contig2	glutamine synthetase	*GS2*
	EX767226	squamosa promoter-binding-like protein 13	*SPL13*
	FD556392	squamosa promoter-binding-like protein 2	*SPL2*
	FD946000, FD946993, FD946993, FD560558, FD988312, EY898725, EX756914, EV551892, EV528056, Rsa#S43017568	squamosa promoter-binding-like protein 3	*SPL3*
	FD557561, EX762240	squamosa promoter-binding-like protein 5	*SPL5*
	EY930450	squamosa promoter-binding-like protein 6	*SPL6*
	EX886942, EW715846,EX771535, EV548910	squamosa promoter-binding-like protein 9	*SPL9*
miR159	EY949798, EY938664, Rsa#S42037487	myb domain protein 101	*MYB101*
	EY896930	myb domain protein 65	*MYB65*
	FD584389, EY935636, FD977876, Rsa#S42034459, Rsa#S42591074	putative ubiquitin-conjugating enzyme E2 17	*UBC17*
miR160	FD576484, FD550653, EV524607, Rsa#S42581764	auxin response factor 16	*ARF16*
	EX896877	auxin response factor 17	*ARF17*
miR164	EX773809	NAC domain containing protein 80	*NAC80*
	EW715661, EV566600	NAC domain containing protein 100	*NAC100*
miR165/166	Unigene8382	defensin-like protein 2	*LCR69*
	Unigene16151	ATP synthase subunit G protein	
miR169	EV547500, EV543544	glutamate decarboxylase 5	*GAD5*
	Unigene27845	histone acetyltransferase HAC1	*HAC1*
	EX750227	nuclear transcription factor Y subunit A-2	*NF-YA2*
	FD989248, EV526819	nuclear transcription factor Y subunit A-3	*NF-YA3*
miR172	EX761783	AP2-like ethylene-responsive transcription factor SNZ	*SNZ*
	EW732550	AP2-like ethylene-responsive transcription factor TOE2	*TOE2*
	EY906836, FD572123	Floral homeotic protein APETALA 2	*AP2*
	EY910663	sulfate transporter 1.3	*SULTR1;3*
miR395	EX903518, FY437914, FY449298, FY448599, FY444096, FY449933, FY444103, EV539245	ATP sulfurylase 1	*APS1*
	FY443904, FY441630, FY445587, FY444933,FY443821, FY442807, FY442799, EY933376, EY916371	sulfate adenylyltransferase	*APS4*
miR396	EW733962, EY907316	transcription factor bHLH74	
	EW733484, FY442946, FD941766, FD563793, EW713404, EW713403, FD971887, FD555982, FD551358, FD541643, EY908741, EX771641, EV550709, EV536225, EV535300, EV528362, EV524610	atypical CYS HIS rich thioredoxin 5	*ACHT5*
	EY930318	L-ascorbate peroxidase 1	*APX1*
miR397	FY438692	laccase 2	*LAC2*
	FD987950, FY452752	laccase 11	*LAC11*
	FD989705, EV528485	laccase 17	*LAC17*
	Unigene4183	Lectin-domain containing receptor kinase A4.3	*LECRKA4.3*
miR398	FD972015, FD549426, FD544311, EX773977, EX757683, EX749645, EV525782	superoxide dismutase [Cu-Zn]	*CSD1*
miR399	FD560927, EX890146	putative ubiquitin-conjugating enzyme E2 24	*PHO2/UBC24*
miR403	EW726356, EX749374, Rsa#S41987411	Argonaute family protein	*AGO2*
miR414	Unigene3598	ABC transporter G family member 10	
	FD967208	AP2-like ethylene-responsive transcription factor SNZ	*SNZ*
	CL5793.Contig1	CBL-interacting serine/threonine-protein kinase 21	*CIPK21*
	Unigene10439	pentatricopeptide repeat-containing protein	
	EV527283, EY913653	potassium transporter	*KUP3p*
	EX886639	scarecrow-like protein 13	*SCL13*
miR482	FD580320	MATE efflux family protein	*FRD3*
miR841	Unigene15520	bZIP transcription factor family protein	

Detailed information of target genes for all salt-responsive miRNAs was listed in Additional file 5.

Many of the predicted target genes were homologous to those encoding some stress-related transcription factors (TFs), including SPB-like proteins (*SPLs*), myb domain proteins (*MYBs*), auxin response factor (*ARF*) family, APETALA2 (*AP2*), NAC domain-containing proteins (*NACs*), nuclear transcription factor Y (*NF-YA* and *NF-YB*) and *bZIP* (Table 6 and Additional file 5). Moreover, several target genes encoding important enzymes or functional proteins playing roles in diverse metabolic pathways, such as argonaute (*AGO2*), glutamine synthetase (*GS2*), glutamate decarboxylase 5 (*GAD5*), argininosuccinate synthase, S-adenosylmethionine (SAM)-dependent methyltransferase, pentatricopeptide repeats (*PPRs*) and histone acetyltransferase (*HAC1*), were also identified. By annotation of targets, a few transcripts were found likely to participate in plant abiotic stress responses. For instance, a miR414 target was CBL-interacting serine/threonine-protein kinase 21 (*CIPK21*), which was related to stress signal perception and transduction [32]; miR397a targeted laccases (*LACs*), which were involved in lignification and thickening of the cell wall [33]. In general, these results implied that these putative target genes might be implicated in diverse biological processes under salt stress in radish.

### RT-qPCR validation

**Figure 6:**
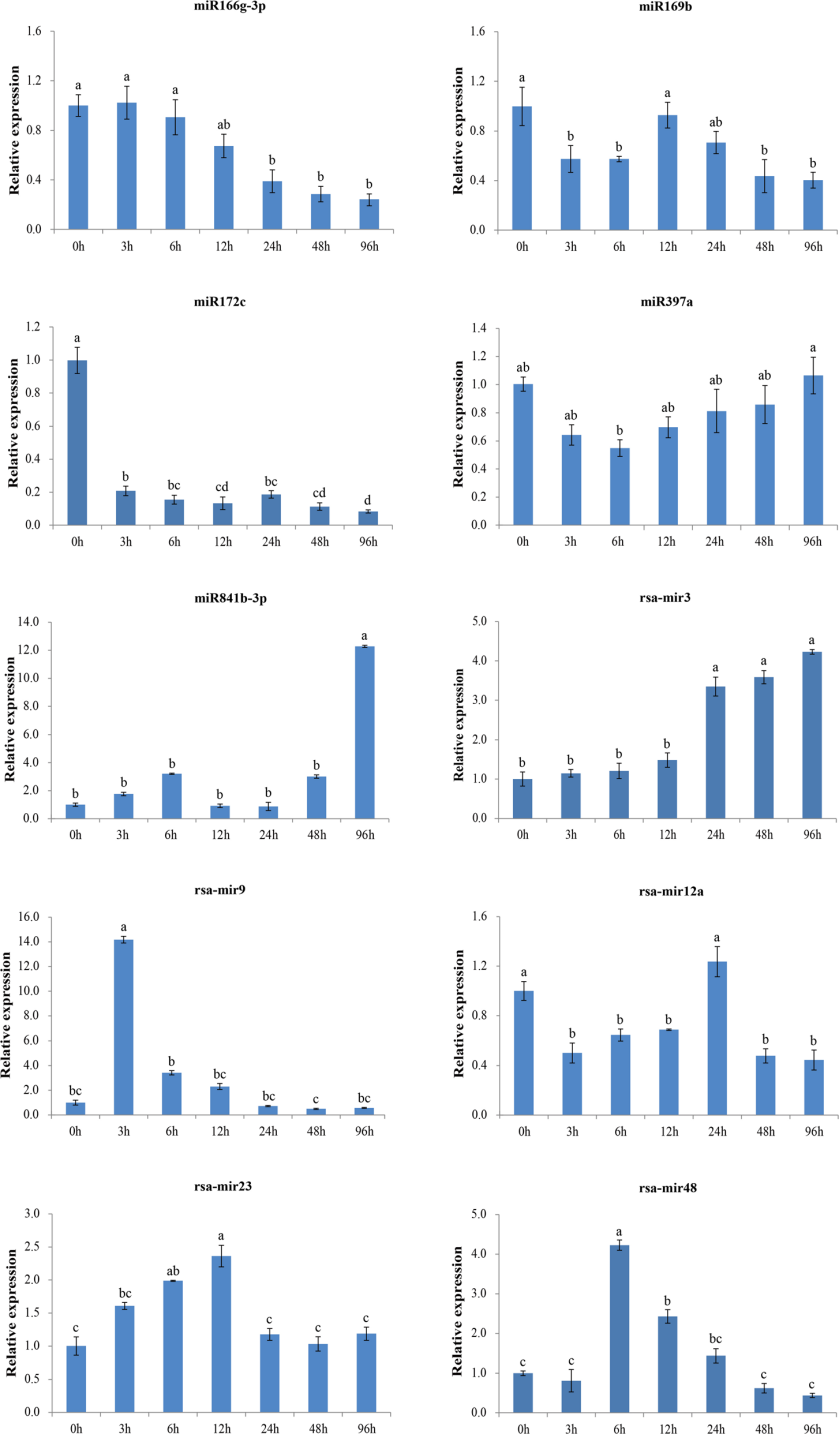
**RT-qPCR analysis of several salt stress-responsive miRNAs.** The expression level in the untreated sample (0 h) was set to a value of 1. Each bar shows the mean ± SE of triplicate assays.

To verify the results of deep sequencing and detect the dynamic expression profiles of salt-responsive miRNAs at different stages of salt treatment (0, 3, 6, 12, 24, 48 and 96 h), the expression of ten salt-responsive miRNAs were analyzed with RT-qPCR (Figure 6). As expected, the obtained data suggested that all examined miRNAs shared a coincidental expression change between sRNA sequencing and RT-qPCR. For known miRNAs, miR166g-3p had a downregulated expression pattern, except that it slightly increased at 1 h of salt treatment. The expression of downregulated miR169b fell at first then abruptly increased at 12 h, and again gradually decreased to a low level. In contrast to miR169b, miR841b-3p showed an opposite expression trend with increasing treatment time. miR172c was downregulated and maintained at a quite low level at all treatment stages. With the increase of treatment time, the expression of miR397a was initially restricted, but then gradually restored to the initial level. For the novel miRNAs, rsa-mir3 expression increased until 24 h of salt treatment. rsa-mir9 and rsa-mir48 shared a similar expression pattern, but they reached their maxima at 3 h and 6 h, respectively. rsa-mir12a was downregulated at nearly all treatment stages, except that it abruptly elevated at 24 h. The level of rsa-mir23 gradually increased to its maximum at 12 h, and then dramatically restored to the initial level.

**Figure 7:**
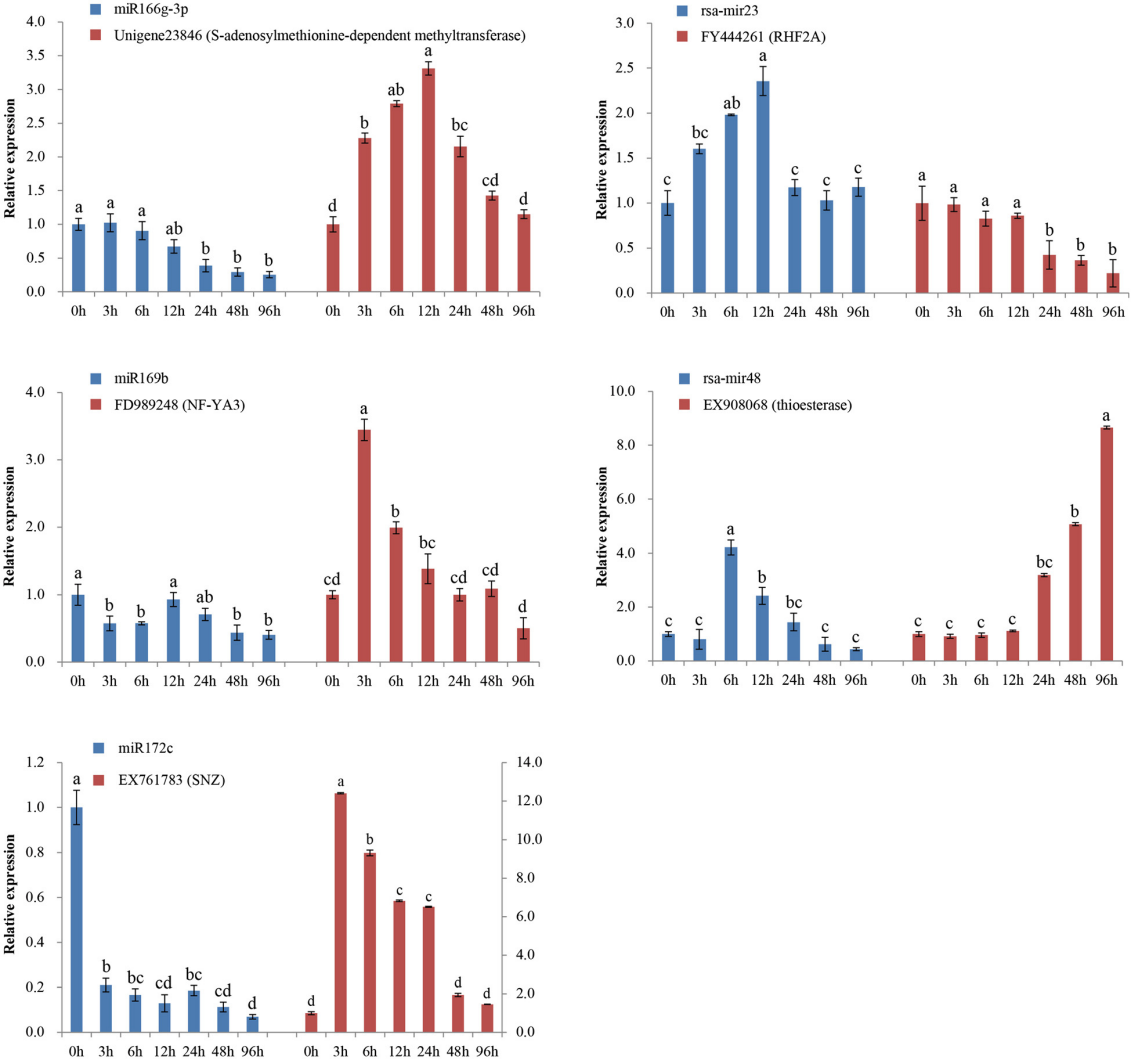
**RT-qPCR analysis of several miRNA-targets.** The expression level in the untreated sample (0 h) was set to a value of 1. Each bar shows the mean ± SE of triplicate assays. Blue and red represent miRNA(s) and their corresponding target gene(s), respectively.

Furthermore, the expression patterns of five corresponding target genes, namely SAM-dependent methyltransferase gene (unigene23846 targeted by miR166g-3p), *NF-YA3* (FD989248 targeted by miR169b), *SNZ* (EX761783 targeted by miR172c), *RHF2A* (FY444261 targeted by rsa-mir23) and thioesterase gene (EX908068 targeted by rsa-mir48), were also examined to confirm the dynamic correlation between the miRNAs and their target genes under salt stress. The results revealed an approximately negative correlation between the expression of miRNAs and their corresponding targets (Figure 7). For instance, the salt-stressed downregulation of miR166g-3p and miR172c led to upregulation of SAM-dependent methyltransferase gene and *SNZ*, respectively. However, the expression of *RHF2A* was found to be restrained by upregulated rsa-mir23 in the early stage of salt exposure.

## Discussions

As one of the most serious abiotic stresses worldwide, salt stress poses an increasingly severe threat to plant growth and development. In plants, to cope with salt stress, the modulation of numerous stress-responsive genes at transcriptional and posttranscriptional levels is activated. MicroRNAs are well known as ubiquitous regulators of gene expression and play vital roles in plant responses to abiotic and biotic stresses [34]. In recent years, increasing reports have demonstrated that miRNA-guided gene regulation plays a significant role in salt stress response in various plant species, such as *Arabidopsis* [7], *P. tomentosa* [8], soybean [19] and *S. linnaeanum* [20]. Radish is an important root vegetable crop worldwide. Although several studies of miRNAs associated with some important horticulture traits have been conducted in radish [25-27], no study on identification of salt-responsive miRNAs and their target genes has been reported to date.

### sRNA sequencing and identification of known miRNAs in radish

In the present study, a total of 3.37 M and 4.20 M unique sRNA sequences were obtained from CK and Na200 libraries, respectively, suggesting adequate sequencing depth for further analysis. 21 nt sRNAs might play more prevailing roles in salt stress response due to their most abundant expression in both libraries (Figure 1), which was consistent with previous studies in several tree and vine species including *Populus* [8,29], trifoliate orange [13], and grapevine [30]. In addition, a striking divergence also existed in expression patterns among diverse types of sRNAs, which showed that the levels of 21 nt and 22 nt sRNAs were markedly decreased under salt stress, whereas those of 20 nt, 23 nt and 24 nt sRNAs were significantly increased (Figure 1). Due to the identified sRNAs with different sizes represented diverse functions in regulating gene expression, a more extensive modulation of gene expression by sRNAs might exist during response to salt stress.

The diversity of radish miRNA families could be determined by the abundance and number of members. In this study, conserved miRNA families had relatively higher expression and number of family members when compared with non-conserved ones (Figures 2 and 3), which was in agreement with previous findings in radish [25,26]. Moreover, it was also inferred that the read number of miRNAs varied from one to millions of copies reflected their diverse expression levels in radish. For example, miR156/157, miR158, miR166, miR168 and miR408 had extraordinarily high number of reads, suggesting these miRNAs might be expressed at a higher level, whereas miR161 and miR393 showed low abundance with less than 100 reads, therefore, possibly were expressed at a lower level.

### Characterization of salt-responsive miRNAs in radish

A number of miRNAs were regulated by salt stress in diverse plant species, such as *Arabidopsis* [7], *Z. mays* [15] and *S. linnaeanum* [20]. In *Arabidopsis*, several upregulated miRNAs (miR156, miR158, miR159, miR165, miR168, miR169, miR171, miR319, miR393, miR394, miR396 and miR397) and downregulated miR398 were detected under salt stress using the miRNA-microarray technology [7]. By the same method, Ding et al. [15] also reported that miR156, miR164, miR167 and miR396 were downregulated, while miR162, miR168, miR395 and miR474 were upregulated in salt-stressed *Z. mays*. Moreover, by NGS technology, 11 downregulated miRNAs (miR156b/c, miR162b, miR167a/b, miR171b/e, miR172a, miR319a, miR399b and miR5300) and three upregulated miRNAs (miR164c, miR166d and miR397a) were identified in *S. linnaeanum* under NaCl exposure [20]. Many miRNAs were evolutionarily conserved across different plant species, which indicated that the conserved regulation of miRNAs played a vital role in plant response to salt stress. In the present study, 49 known miRNAs belonging to 28 miRNA families were differentially expressed and considered as salt-responsive miRNAs (Figure 4a and Additional file 4). However, several salt-responsive miRNA families including miR156, miR168, miR319, miR391, miR403 and miR857, did not show significant alterations of expression in the presence of salt stress in radish, although some of them were detected to be significantly altered by salt stress in other plant species [7,15]. This discrepancy suggested that these miRNAs potentially expressed in a species-specific manner under salt stress.

Additionally, it was worth noting that some salt-responsive miRNAs identified in this study might be fine-tuned across distinct biotic and abiotic stresses. For instance, miR169 and miR319 were widely reported to play important roles in ABA, drought and salt stress responses [4,35,36], and miR398 was considered as a bridge linking plant responses to oxidative stress and other stresses such as water deficit, salt stress, ABA stress, UV stress, nutrient deficiency and bacterial infection [35,37]. This observation was likely to be attributed to the shared regulatory genes modulated by these stress-related miRNAs across diverse stress responses, indicating that the inferred cross-regulation of miRNAs might link plant responses to various stresses [4]. However, further efforts are still needed to precisely confirm the roles of these salt-responsive miRNAs and explore the regulatory mechanism underlying these functions in plant adaptive response to salt stress.

### miRNA-mediated regulatory network of salt stress response in radish

**Figure 8:**
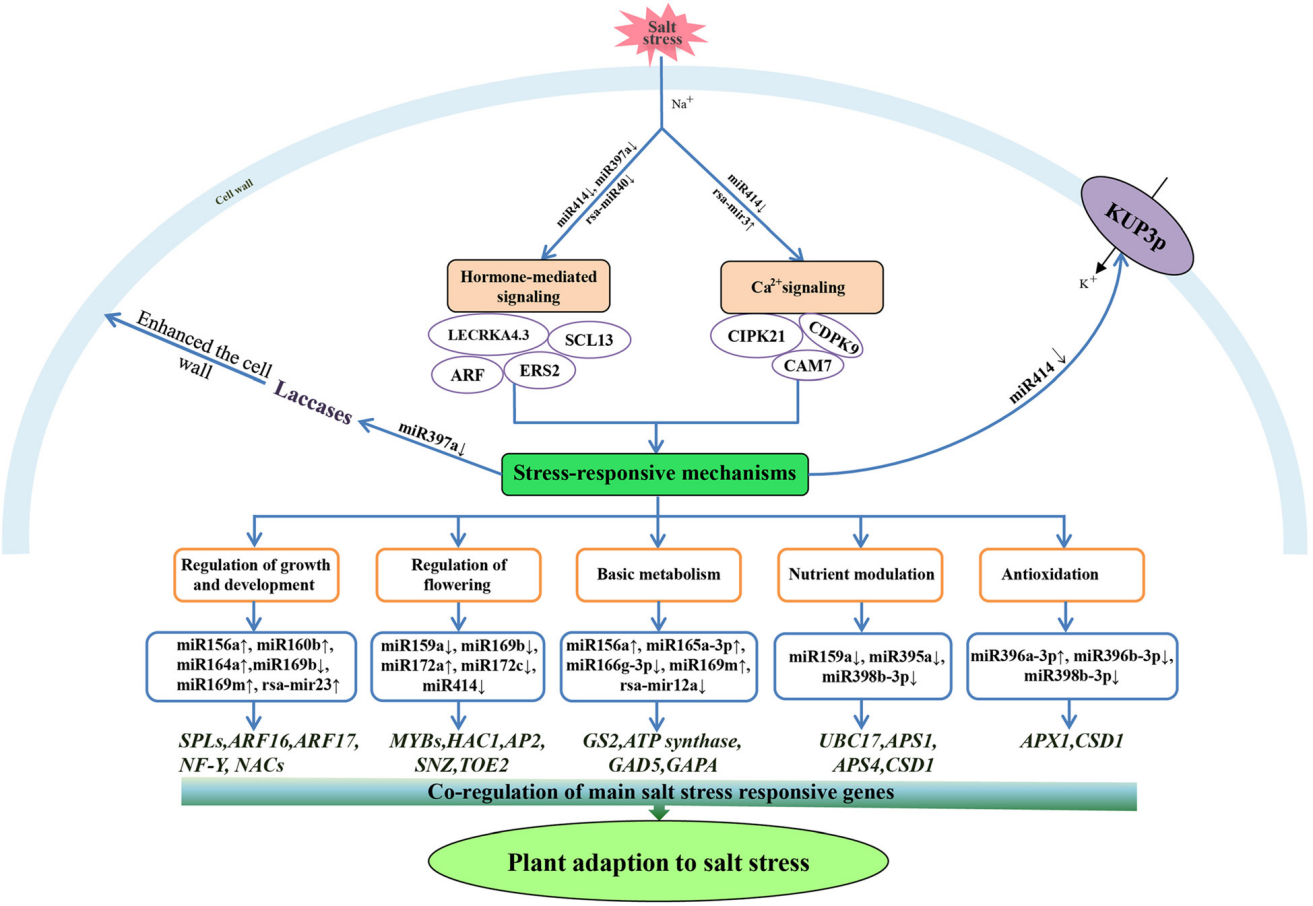
**The proposed model of miRNA-mediated regulatory network associated with salt stress response in radish.** The potential interactions between salt-responsive miRNAs and their corresponding target genes were shown.

microRNAs function in gene modulation by regulating specific mRNA transcripts for degradation in plants. Recently, the NGS technology coupled with bioinformatics analysis have been widely applied to identify miRNAs and their corresponding target genes responsive to salt stress in plants [8,16,18,20]. In this study, a number of miRNAs were identified to be salt-responsive in radish, and many of them might play crucial roles in regulatory network of salt stress response by regulating specific stress-related genes. On basis of these results, a putative model of miRNA-target interactions involved in plant response to salt stress was put forward here, which presented the proposed regulation cascades after salt exposure in radish root cells (Figure 8).

In the present study, target prediction for 65 salt-responsive miRNAs revealed that many targets were TF genes including *SPLs*, *MYBs*, *ARFs*, *AP2*, *NACs* and *NF-Y* (Table 6 and Figure 8), which were reported to function in activating stress-responsive genes [38]. Many target genes might play important roles in plant responses to abiotic stresses. For example, miR169-targeted nuclear factor Y subunit A (*NF-YA*), which conditioned whole plant growth through modifying carbohydrate metabolism and cell elongation, was widely regulated under drought and salt stress [36,39]. Moreover, a set of evidences also supported the involvement of miR164 in stress responses, regulating the miRNA-mediated cleavage of *NAC. NACs* were widely modulated by various abiotic stresses like salinity, cold, ABA or drought [40-42], and also integrated responses to environmental stimuli into regulation of plant development processes [43-45].

Additionally, different members of auxin response factors (ARF) family (*ARF16* and *ARF17*), which were reported to participate in auxin signaling pathways and be negative regulators of growth and development [4,46], were also identified for salt-responsive miR160b in radish. Similar findings of miR160-mediated ARF regulation were also reported in salt-stressed *P. tomentosa* [8], drought-stressed *P. persica* [10] and cold-stressed trifoliate orange [13]. These observations implied miR160-regulated *ARFs* might play an important role in plant responses to various abiotic stresses by fine-tuning plant growth and development under stress conditions. Other genes including miR156/157-targeted *SPLs* (*SPL2*, *SPL3*, *SPL5*, *SPL6*, *SPL9* and *SPL13*) and miR172-targeted *AP2*, which were involved in regulating plant growth and development, were also identified in this study. It was reported that miR156-regulated *SPLs* and miR172-targeted *AP2* conjointly conditioned the transitions among different developmental stages including embryogenesis, vegetative and reproductive growth, and participated in determining floral organ identity [47,48]. Similar to *SPLs* and *AP2*, miR159-regulated *MYBs* (*MYB65* and *MYB101*) were also considered to modulate plant growth and development especially flowering under salt stress. It was reported that overexpressed miR159 resulted in a delayed flowering state concomitant with a repression of its target gene, *GAMYB* in gloxinia [17]. Given that plant growth and development including bolting and flowering were usually adversely-influenced under stress conditions, it might indicate the involvement of these miRNA-target transcripts in the network of genes regulated by salt-responsive miRNAs in radish (Figure 8).

Apart from key TFs, a number of genes which encode important enzymes or functional proteins, such as APX1, CSD1, APSs (APS1 and APS4), LACs (LAC2, LAC11 and LAC17), UBCs (UBC17 and UBC24), Ca^2+^-mediated signal-related proteins (CAM7, CIPK21 and CDPK9), were also considered to play important roles in salt stress response. Among them, *CIPK21*, *CAM7* and *CDPK9* (targeted by miR414, rsa-mir3 and rsa-mir5, respectively) were reported to cooperatively perform their functions to initiate the stimulus-specific downstream signal transduction [32]. miR397-targeted *LACs* encoding laccases were related to lignification and thickening of the cell wall in secondary cell growth [33], and accordingly enhanced the ability to alleviate stress damage. Furthermore, several target genes were found to be cross-regulated by miRNAs in response to salt stress and other abiotic stresses including oxidative stress and nutrient stress. As represented in this study, miR398b-3p targeted *CSD1* encoding superoxide dismutase [Cu-Zn], which was reported to participate in scavenging excess reactive oxygen species (ROS) in plants exposed to salt stress [49]. Additionally, L-ascorbate peroxidase encoded by miR396-regulated *APX1* was also a significant ROS scavenging enzyme, which was involved in regulation of intracellular ROS expression levels [50]. Several similar miRNA-targets had also been identified in plants exposed to nutrient deficiency. For instance, miR395 was widely reported to participate in catalyzing inorganic sulfate assimilation by suppressing the expression of ATP sulfurylases (*APSs*) [51], and miR398-mediated *CSD1* regulation was responsible for maintaining copper concentration in plant cells [52]. Moreover, miR399 was also found to regulate phosphorus homeostasis by modulating the expression of ubiquitin-conjugating E2 enzyme (*UBC*) [53,54]. On account of the fact that prolonged salt stress could usually lead to some secondary stresses such as oxidative stress and nutrition disorder [55], the results indicated that these miRNA-regulated target genes might play significant roles in plant adaptive response to salt stress, as an indispensable part of regulatory network responsive to salt stress in radish (Figure 8).

## Conclusions

The application of sRNA sequencing technology combined with bioinformatics analysis provides an unprecedented opportunity to obtain comprehensive understanding of novel and salt-responsive miRNAs in radish. A total of 49 known and 22 potential novel miRNAs were differentially expressed under salt stress. Prediction and analysis of target genes for these salt-responsive miRNAs demonstrated that numerous transcription and regulatory factors (enzymes) primarily functioned in a variety of biological pathways under stress conditions, including stress-related signal sensing and transduction, regulation of ion homeostasis, basic metabolic processes, secondary stress responses, as well as modulation of attenuated plant growth and development. Given that plant responses to abiotic stresses consist of many complex biochemical processes in which different components operate together, this investigation could advance our insights into the miRNA-mediated regulatory network of salt stress response, and the results will facilitate further dissection of molecular mechanism underlying plant response to salt stress in radish and other root vegetable crops.

## Methods

### Plant materials and salt stress treatment

The seeds of advanced inbred line “NAU-YH” were surface-sterilized with 1% sodium hypochlorite, and then germinated at 25°C for 2 d in the dark. Germinated seeds were cultured in individual pots containing loam soil and grown in a growth chamber with a 16-h light at 25°C and 8-h dark at 18°C cycle. Three-week-old seedlings with thickened flesh roots were transferred into modified half-strength hoagland nutrient solution as previously described [56]. For the salt-treated group, the radish seedlings were treated with 200 mM NaCl for 3 h, 6 h, 12 h, 24 h, 48 h and 96 h, respectively. Seedlings cultured under NaCl-free solution were used as control. Fresh roots at different salt-treated time points were immediately collected and stored at −80°C for further use.

### Small RNA (sRNA) library construction and sequencing

Total RNA was extracted from the control (NaCl-free, CK) and salt-stressed (200 mM NaCl for 48 h, Na200) radish roots with TRIzol reagent (Invitrogen, USA) following the manufacturer’s instructions. Two sRNA libraries were constructed according to previously reported procedures [25,57]. Briefly, sRNA fractions of 18–30 nt isolated and purified by 15% denaturing polyacrylamide gel electrophoresis were ligated with specialized adaptors to the 5’ and 3’ ends (Illumina) using T4 RNA ligase. They were then reverse transcribed to cDNA using SuperScript II Reverse Transcriptase (Invitrogen), followed by PCR amplification. The final PCR products were purified and subjected to deep sequencing using Solexa sequencer (Illumina) HiSeq2000 at the Beijing Genomics Institute (BGI), Shenzhen, China.

### Identification of known and novel miRNAs

After removing undesirable raw reads including low-quality reads, adapter reads, contaminants and reads either shorter than 15 nt or longer than 30 nt, the remaining unique sequences were mapped to the radish reference genome which consisted of radish GSS, EST and transcriptome sequences, to analyze the expression and distribution of sRNAs on genome using SOAP2 program [25,26]. Perfectly matched sequences were retained for following analysis. By querying against the NCBI Genbank (http://www.ncbi.nlm.nih.gov/genbank/) and Rfam (10.1) (http://www.sanger.ac.uk/resources/databases/rfam.html) databases, the sRNA sequences matching rRNA, tRNA, snRNA, snoRNA as well as sequences containing poly (A) tails were excluded. The remaining unique sequences were aligned against miRBase 20.0 (http://www.mirbase.org/index.shtml) to identify radish known miRNAs. The matched sequences with no more than two mismatches were considered as known miRNAs. Then, unannotated unique sequences were mapped to the radish reference sequences to uncover novel miRNAs from radish, according to previous criteria [31], using MIREAP software (http://sourceforge.net/projects/mireap/). The sRNA secondary structures were also formed by Mfold (http://mfold.rna.albany.edu/?q=DINAMelt/Quickfold).

### Differential expression analysis of miRNAs responsive to salt stress

The counts of identified miRNAs in two libraries were normalized as transcripts per million (TPM) according to the formula: Normalised expression = actual miRNA count/total count of clean reads × 1,000,000. The normalized values of miRNAs with abundance of zero were modified to 0.01 for further analysis. The remaining normalized reads were used to calculate the *p*-value and the change in expression abundance. The differential expression of miRNAs between two libraries was calculated as: Fold-change = log_2_ (Na200/CK). The *p*-value was obtained according to previously reported methods [19]. The miRNAs with values of log_2_ ratio (Na200/CK) > 0.5 and < −0.5, along with the *p*-value of < 0.05, were considered as upregulated and downregulated during salt stress, respectively.

### Prediction of putative miRNA targets for salt-responsive miRNAs

The putative target genes were predicted by the plant small RNA target analysis server as described by Barvkar et al. [58] and Zhai et al. [26]. The criteria for target prediction were based on previous studies following the alignment between each miRNA and their targets [59,60], including: (1) no more than four mismatches; (2) no more than two adjacent mismatches in miRNA/target duplex; (3) no more than one mismatch at positions 1–9; (4) no mismatches at positions 10–11, and (5) no more than 2.5 mismatches in positions 1–12 of the miRNA/target duplex (5’ end of the miRNA). To understand the biological functions of the target genes, the Blast2GO program was applied to obtain the GO annotations on the basis of the BLAST searches against the available Nr database in NCBI [24,26,61].

### Validation of miRNAs and their potential target genes by RT-qPCR

Small RNAs were extracted from samples exposed to 200 mM NaCl at different time points (0, 3, 6, 12, 24, 48 and 96 h) using a small RNA isolation kit (TaKaRa, Dalian, China) according to the manufacturer’s instructions. Then, the small RNAs were reverse-transcribed to cDNAs using the One Step PrimeScript® miRNA cDNA Synthesis Kit following the manufacturer’s protocols. For RT-qPCR validation of target genes, total RNAs were isolated as described above and reverse-transcribed into first-strand cDNA using the Superscript III First-Strand Synthesis System (Invitrogen, USA). The primer sequences of miRNAs and their target genes were shown in Additional file 6. The RT-qPCR analysis was carried out on an iCycler iQ real-time PCR detection system (BIO-RAD) using SYBR® Premix Ex Taq™ II (TaKaRa). Each PCR reaction was carried out in a total volume of 20 μl containing 2 μl of cDNA, 10 μl of 2 × SYBR Green PCR Master Mix, and 0.2 μM primer pairs. The PCR amplified condition was performed following the previous reports [25,26]. The 5.8S rRNA was used as the internal reference gene for normalization. All reactions were run in triplicate and the data were statistically analyzed by SAS Version 9.0 software (SAS Institute, NC, USA) using Duncan’s multiple range test at the *P* < 0.05 level of significance.

## Additional files

Additional file 1:
**The detailed information of known miRNAs identified in radish.**
Additional file 2:
**The detailed information of novel miRNA candidates identified in radish.**
Additional file 3:
**The secondary structures of identified potential novel miRNAs in radish.**
Additional file 4:
**Summary of differentially expressed miRNAs under salt stress in radish.**
Additional file 5:
**Targets and functional annotations for the salt-responsive miRNAs in radish.**
Additional file 6:
**The miRNAs, targets and their primer sequences for RT-qPCR validation.**

## Abbreviations

miRNA: MicroRNA
SPL: Squamosa promoter binding-like protein
ARF: Auxin response factor
NF-Y: Nuclear transcription factor Y
RT-qPCR: Reverse-transcription quantitative PCR
RISC: RNA-induced silencing complex
NGS: Next generation sequencing
EST: Expressed Sequence Tag
GSS: Genome survey sequence
MFE: Minimum free energy
GO: Gene Ontology
MYB: Myb domain protein
AP2: APETALA2
LAC: Laccase
ROS: Reactive oxygen species
snRNA: Small nuclear RNA
snoRNA: Small nucleolar RNA
TPM: Transcripts per million
