# Supplementary material for: Identification of novel and salt-responsive miRNAs to explore miRNA-mediated regulatory network of salt stress response in radish (Raphanus sativus L.)
Source: BMC Genomics. 2015 Mar 17;16(1):197. doi: 10.1186/s12864-015-1416-5 (PMC4381364; doi:10.1186/s12864-015-1416-5)
Supplement: Additional file 3: — The secondary structures of identified potential novel miRNAs in radish. [file 12864_2015_1416_MOESM3_ESM.pdf]

Additional file 3: The secondary structures of identified potentially novel miRNAs in radish. (“.” represent base mismatches, “(” represent base matches)

rsa-mir1

NAU_YH_CK-m0036 Unigene29179_All:31:258:+ 228(nt) -74.20 (kcal/mol)

UAGGGUUCGUAAAGGUUUAGGAUUUAGGGUUCGUAAAGAUUAAGGGUUUAGGGUAUAGGGUUCGUGUAAAGGGUUUAGGGUUC
GUGAACCCUAUGGGUUUGGAGUUUAAGGUUUGUGAACCUAAGGGUUUGGGGUUUAGGGAAGGGUUUGGGGUUUAGGGUUCGUG
AACCCUAAGGGUUUAGGGUUUAGAGUUCGUGAACCCUAAGGUUUUAGAGUUCGUGAACCCUU

.....(((.(.(((.((((((.(((.(((((((((((..(((((..((((((.((((((((((((................)
))))))))))))..)))))).)))))..))))))))))).))).)))))).))).).)))............(((((((((
(((.((((((.(((((((((((.......))))))))))).)))))).))))))))))))).

**********AAAGGUUUAGGAUUUAGGGUU***
****
****

rsa-mir2

NAU_YH_NaCl-m0004 CL10794.Contig2_All:1244:1428:+ 185(nt) -71.30(kcal/mol)

CAAAGAAAAGAAAUCAUACUUUCAUUGAUAAUGAAAUCAAUAGAGAGAAUGAUAAUGAGCUCUUACAUGCAAUUCAAAA
AUAUAGUAUCUCUUAGAUCCUCUAAUUGCUUGUAAGAGUUGAUGACCAAAUGGGUUAUCCUCUAUUGAUUUCAUCAUCA
AUGAAAGGUAUGAUUCCCUUUUUUUGA

(((((((.(.((((((((((((((((((.(((((((((((((((...........(((((((((.((((((.....
.....((.((((...)))).))..)))))).))))))))))((((((.....))))))))))))))))))))))).))))
)))))).)))))))).).)))))))).

**********AAAUCAUACUUUCAUUGAUA**
***UCA
AUGAAAGGUAUGAUUCCC*********

rsa-mir3

NAU_YH_NaCl-m0016 CL4756.Contig1_All:969:1086:- 118(nt) -66.70(kcal/mol)

CUUGGUCAAGUGUAAUGUAUGUAGUCCAAUCUAUUGAAGUACUAGCACACCAUCUCUAGUGAGGUGAUGUGGUUUGAGUAGUU
CAAUACAUUGGACUACAUAUAUUACAUUUGAUCAA

.(((((((((((((((((((((((((((.(((((((.(((((.((((.((((((....)))))).)))).)...)))).))
))))).)))))))))))))))))))))))))))

****
****ACAUUGGACUACAUAUAUUAC**********

rsa-mir4

NAU_YH_NaCl-m0113 gi|332787496|gb|FY453420.1|FY453420:315:421:+ 107(nt) -64.50(kcal/mol)

AUUGAUGCGUUUGACGUUUCUCGAACUCAAGACCUCAAUUGGUGACAACUUGUCCAGAGUUGUUAAAAUGGUGAGGUCUUGAG
UUCGAGGGACGCCAAGCGCACCGA

.(((.((((((((.(((((((((((((((((((((((.....(((((((((.....)))))))))......))))))))))))
)))))))))))).)))))))).)))

*************ACGUUUCUCGAACUCAAGACC***************************************UCUUGAG
UUCGAGGGACGCCA**********

rsa-mir5
NAU_YH_NaCl-m0012 CL2420.Contig1_All:387:566:+ 180(nt) -48.10(kcal/mol)
AGCAUGAUGGGAAUCCGCUCCUGGUAUGCAGAACUUGUUCUUUGUGUAUCGUCAGGCGCCGAAUACGUUGCGUAUUACCAGUU
UCUUUGUCAAACUACGUUCUCCAUUGGUGGAGCAACUUUUGUCUUCUCAGGUAACAGUGUGCGUGUACUGUUGGGGAUGGAUU
GGCACCAUCCACAA
.....((((.........((((((((..(((.(((((...((((.......)))).))))).)))..))).))))))..
....((((((.(((...((((((.(((((.(((((((.((((((....)))))).))).))).).))))).)))))))))).))
))))))))).....
****
***ACUGUUGGGGAUGGAUU
GGCA**********

rsa-mir6
NAU_YH_NaCl-m0060 gi|156155651|gb|EW715797.1|EW715797:300:370:+ 71(nt)
-31.20(kcal/mol)
AUCGCAGUGGUUCUUCUGGCCGACGGCGGUGGGCUUAUACAGACACGUCGUCGGUCGGGAUUUAUUGUCGG
.((((((((...((((((((((((((((..(......)..)).))))))))))))))).))))))).)))
AGACACGUCGUCGGUCGGGAU********

rsa-mir7a
NAU_YH_NaCl-m0049 gi|154172381|gb|EV543654.1|EV543654:457:605:- 149(nt)
-41.50(kcal/mol)
AUAGCGGUUCUUGAUUUGUUAAGCUUCUUUGCUUCUCGAAUCUCGAUAGAUCUUGGCCUCGCAAAAUGAUUUCCUUGAA
AACAAAGAACUGGAAGUAGGGCUCCUUAUAGUUCAAACGAGAGUUUAGCGGGGUAAAGAAAAGCUGCUAA
.(((((((((..((((((..((((......))))..))))))..))....((((.(((((((.((((..(((..(((((
.....((..(((....)))..))........)))))..))).)))).))))))).))))...))))))).
****
**************************************AGAGUUUAGCGGGGUAAAGAA**********

rsa-mir7b
NAU_YH_CK-m0048 gi|154172381|gb|EV543654.1|EV543654:456:606:- 151(nt)
-41.50(kcal/mol)
AAUAGCGGUUCUUGAUUUGUUAAGCUUCUUUGCUUCUCGAAUCUCGAUAGAUCUUGGCCUCGCAAAAUGAUUUCCUUGA
AAACAAAGAACUGGAAGUAGGGCUCCUUAUAGUUCAAACGAGAGUUUAGCGGGGUAAAGAAAAGCUGCUAAG
..(((((((((..((((((..((((......))))..))))))..))....((((.(((((((.((((..(((..((((
(.....((..(((....)))..))........)))))..))).)))).))))))).))))...)))))))..
****
***************************************AGAGUUUAGCGGGGUAAAGAAA**********

rsa-mir8
NAU_YH_NaCl-m0015 CL4175.Contig1_All:1:195:- 195(nt) -43.10(kcal/mol)

ACACAUGUUCGCAUAACCAUCCCAAUUUGUUCCUUGUCCCACAAAAGAGCCGGGUUUUUGACUAUCGCAAAUCGAGCUU
GCAAUACUCCAAAAGCACGCUCCACAUCUUUUCGGGUUGAUUCUUGAUAUUCAGCAAAUAACUCUGCUUUGGGACCUUG
AGGGUGUGAGAUAGAUUGGAUAAAUGUUGACCAUUUU
......(((.((((....((((.((((((((((((((((((.....((((.(((((.((((.(((((..((((((((.(
((...........))).))))...((((....)))).))))..))))).)))).....))))).))))))))))...)
)))......))))))))))))..)))).)))......
****
****
********AGAUAGAUUGGAUAAAUGUUGA*******

rsa-mir9
NAU_YH_CK-m0101   gi|167429570|gb|FD935332.1|FD935332:312:413:-   102(nt)
-46.30(kcal/mol)
CUCUUCCCAUCCCGUUCAGUUCUCCUUUUGCUUCACAUUGGAUCAGUCUCGUCAAGACUGGUUCGAUACGAAGCAAACG
AGAAUUGAACGGAACAAAGCACA
...........((((((((((((..((((((((..((((((((((((.....))))))))))))..)))))))).)
))))))))))))..........
**AGCAAACG
AGAAUUGAACGGA**********

rsa-mir10
NAU_YH_NaCl-m0014 CL3310.Contig5_All:114:196:+ 83(nt) -41.20(kcal/mol)
ACCGAGGAAACCAAGAAAUGGGGUUUCUUCGAAAUUUCCUCACUAUUUUUGAGGAAAUUUCGAGGAAGACCAACUUCC
UCGG
.((((((((.(((.....)))(((((((((((((((((((((........))))))))))))))))).)))))...))))
))))
AGGAAAUUUCGAGGAAGACCA***
****

rsa-mir11
NAU_YH_CK-m0010 CL2420.Contig13_All:3733:3823:- 91(nt) -31.80(kcal/mol)
GCUCAUCGCGAGGCGAUGAUGGAUACCGAGAACAUGACCCGAGAGCAGCGUAUGCUCGUCUCGGUAGCGAUGGUUCAAU
CUCGCGCAGACG
......(((((((.(((.((.(.((((((((.(.........(((((.....))))))))))))).).)).)))...)
))))))......
**********AGGCGAUGAUGGAUACCGAGAA****************************CUCGGUAGCGAUGGUUCAAU
CUCG********

rsa-mir12a
NAU_YH_CK-m0108   gi|167473296|gb|FD958546.1|FD958546:335:445:+   111(nt)
-44.30(kcal/mol)
UCGUGGCUGGCUAGAGAUCUCUUCGAUCUCGAUCUCCUCCUCAGAAUCAGCAAGGGAGGGUGAUUUGUUGCCCGAGGAA
GAGGGGAGGAUGGGUGGGUUUCUCCAGCGACG
.(((.(((((..((((((((.((((.((((..((((.(((((.(...(((((((.........))))))).).))))).

)))).)))).)))).))))))))))))).)))
****
*AGGGGAGGAUGGGUGGGUUUC**********

rsa-mir12b
NAU_YH_CK-m0124 gi|167507240|gb|FD986211.1|FD986211:1:106:- 106(nt)
-42.50(kcal/mol)
UGGCUGGCUAGAGAUCUCUUCGAUCUCGAUCUCCUCUUCAGAAUCAGCAAGGGAGGGUGAUUUGUUGCCCGAGGAAGAG
GGGAGGAUGGGUGGGUUUCUCCAGCGC
..(((((..((((((((..((.((((...((((((((((.......((((.(((......))).)))).....))))))
)))))))).)).))))))))))))))..
**AG
GGGAGGAUGGGUGGGUUUC********

rsa-mir12c
NAU_YH_NaCl-m0097 gi|167473296|gb|FD958546.1|FD958546:335:445:+ 111(nt)
-44.30(kcal/mol)
UCGUGGCUGGCUAGAGAUCUCUUCGAUCUCGAUCUCCUCCUCAGAAUCAGCAAGGGAGGGUGAUUUGUUGCCCGAGGAA
GAGGGGAGGAUGGGUGGGUUUCUCCAGCGACG
.(((.(((((..((((((((.((((.((((..((((.(((((.(...(((((((.........))))))).).))))).
)))).)))).)))).))))))))))))).)))
****
GAGGGGAGGAUGGGUGGGUUUC**********

rsa-mir12d
NAU_YH_NaCl-m0110 gi|167507240|gb|FD986211.1|FD986211:1:106:- 106(nt)
-42.50(kcal/mol)
UGGCUGGCUAGAGAUCUCUUCGAUCUCGAUCUCCUCUUCAGAAUCAGCAAGGGAGGGUGAUUUGUUGCCCGAGGAAGAGGGGA
GGAUGGGUGGGUUUCUCCAGCGC
..(((((..((((((((..((.((((...((((((((((.......((((.(((......))).)))).....))))))))))
)))).)).))))))))))))))..
***GAGGGGA
GGAUGGGUGGGUUUC********

rsa-mir13
NAU_YH_NaCl-m0077 gi|161556551|gb|EY928450.1|EY928450:152:359:- 208(nt)
-33.50(kcal/mol)
UAAAUACUCUAUAUACUGAAGUUUAUACUCUUCAAUCUAGUUAAAAAAUUUCAAACCACAUUGUACAUUUGAAUUUAUG
UACUCUAAACUAUCUUUCAUGUUAUAUAGGAAUUGUAAUAAUUUUUAAUAUUUAUUCUACUAAAGUUUCAUAUAUUGA
CUAGAUUAGUGGAAUUAGGAUCAUAAAAUCUUCAUUAUCUAGAGAAAUGA
......(((((.(((.((((((((((..((((.......((((((((((.............((((((........)))
)))...............((((((.......))))))))))))))))).....((((((((((..(.(((.....)))
..)..))))))))))).)))))..))))))..))))).))).))))).......
**********AUAUACUGAAGUUUAUACUCU***

****
*******************AUCAUAAAAUCUUCAUUAUCUAG********

rsa-mir14
NAU_YH_CK-m0039 Unigene38592_All:6:218:- 213(nt) -36.70(kcal/mol)
CAUAUCACAACUCGGGAUGUUACAGUGACAUGCUCAACUCUCCAUCACUACUUUUCAUGUGUGAAUAUUUCAAGAACAG
AGAAAAUAUAGCUUGGUCAACCAUGAUUAUAGCUAGCUAAAAUAUAUUCUUUCACUCACUAUUUACCUUACAAUUGGGU
UAUACUUUUAGAGGACAUGCAGAAUAAGUUUAGCAUCUAGCGAUCUGAUGAUUCA...((((((..(((((((((((.(
.(....(((....((((...((((.((.......)))))).......(((((..........((((((((((....)
))))).)))))..........))))).............((((......)))).........)))).....)))...
..).).))))))))..)))..)).))))...
*AUAUCACAACUCGGGAUGUUA**
****
****

rsa-mir15
NAU_YH_NaCl-m0001 CL10785.Contig2_All:145:374:- 230(nt) -55.00(kcal/mol)
CUCUUGAGCAUGGGAGUGAAGGUGUACAGUUUGUCAAGGAAAUAGCUUCUCAUUUGCAAAACCUCAGCAUGAAAAAAACA
UAAUCAUACUGAAAUUUGAUGCCGCUUCUACCAGUAAAGAACCUUCGCAAAGAUAACUUUGCGGAGAAGAUAUUGCAGA
UGAAUCUAAUAGAAGCCAAGAGUGGUGAAAAAGCUCAAGGAAUCUGUACAGCAUCUUCUUCAUGGCCUUUUC
........(((((((.((.(.((((((((((.(.........((((((((((((((....((((.((((.......
...)))).))))............((((........)))).((((((((((....))))))))))......)))))))
)))........)))))...((((.........)))).).))).))))))).).))))).)))))........
****
****
AUCUGUACAGCAUCUUCUUCA*******

rsa-mir16
NAU_YH_CK-m0023 CL9006.Contig3_All:198:307:+ 110(nt) -45.50(kcal/mol)
ACGAGAGAGAGACAGGGAACAAGCAGAGCAUGGAUUGAGUUUUACCAAAACAUCAAAUGACUAUGUUUGGUCUCUUCCC
AUGCACUGCCUCUUCCCUGGCUCCCCUCUUUC
..(((((.(((.(((((((...((((.((((((...(((....((((((.(((.........)))))))))))...))
)))).))))...))))))).))).))))))..
****
AUGCACUGCCUCUUCCCUGGC**********

rsa-mir17
NAU_YH_CK-m0015 CL6706.Contig1_All:407:699:+ 293(nt) -95.20(kcal/mol)
AGGCCGGCCGUCCAUCGCCGGAGAAGAAGACAAGGACGCCAGAGACAAAACCAAACGACCACCACAGGUAUGGUAAGAC
GUUGGAGGCAGAGCCGAUCGGAACACCGAAACCGGAAGAAAAAGACACACGGACGAAAGAUCCGACCGACGUCGAGGCU
AAAAAAACCCUCCGACGUCGGUCAGAUUACACAGAUCUUAACCGGAGAAGAUGAGAGAAGUUGGAGCUUUUCGAUAGAG
AGAGAAAACUCGAUACACUGGUUUGCUUGCUGAUUGUUGGGGAUGGAUUGUCACCA
.((..(((.(((((((.((.((.((..((.((((.(.(((((........((((.((...((((......))))....)
))))).(((...)))..((((....))))..((((..............((((....).)))((((((((((((((..

........))).)))))))))))(((((.....)))))...)))).........(((((((....)))))))..((..(
((......)))..))..))))).).))))))..)).)).))))))))).))).)).
****
****
****
********************************AUUGUUGGGGAUGGAUUGUCA***

rsa-mir18
NAU_YH_NaCl-m0085 gi|166119227|gb|FD551622.1|FD551622:586:772:+ 187(nt)
-51.20(kcal/mol)
UGAGGACUGACAGCGCUCUGAAGUUCGGUUGGUUUUCUUCUUCUACCUGAUUCACAUUGGCUUCUGCAUCUUUGCCGC
CAUUGCUCCUCCAAUCUUUUCCGCGGCUUAUCAUUAACGGGUGUGCUGGCAGCAAUUAAUGUAAUCAGAGCCAGUUUA
CUAGCCGGAUCUUCUACUUUGUCGGCUUC
.((((.(((((((.(....(((((((((((((((......((........)).....((((((.(((........(((((
.......................)))))....((((((..(.(((....))).)..))))))....)))))))))))..)
)))))))))).))))..).)))))))))))
**********CAGCGCUCUGAAGUUCGGUUG***
****
****

rsa-mir19
NAU_YH_CK-m0032 Unigene22804_All:1:77:+ 77(nt) -22.10(kcal/mol)
UAAUUUGGGAAGUGGGGGAGAGGGGGGGGAGGUGUUGUAUUAUUCCCUUCUCAUUCGAUGGUCUAGAUGUGCGAGGU
..((((((..((.(((((((((((((.((.........)).))))))))))).))).))..)))))))..........
CCUUCUCAUUCGAUGGUCUAGA*******

rsa-mir20
NAU_YH_NaCl-m0103 gi|167498510|gb|FD981015.1|FD981015:216:349:- 134(nt)
-33.80(kcal/mol)
CUCCUCGCGUCGGAGGAAAACCACCCGGGACACUUGAAGUCUCCUCUGUAGAUCUUCCUAUACAUGUUCACGAGAUUAU
CAUCCUGAAACGGCAAGUAACCAGCGAGGAGCACGUAGAGGAUCACCCCGCAGGA
.((((.(((.(((((((...((....))(((.......))))))))))..(((((((...(((.(((((.((...((((
...((......))...))))....))..))))).))))))))))....)))))))
****
************************CGAGGAGCACGUAGAGGAUCA**********

rsa-mir21
NAU_YH_NaCl-m0019 CL4970.Contig1_All:374:576:- 203(nt) -54.50(kcal/mol)
ACCGGACAUGGCUUACUUCUUCUUUACUAAAGAUAUACUCAGAGGGAAGACGAUUACGGUGUUCGAGUCUCCUGAUAAA
GGUAGUGUCGCUAGGGACUUCACUUACAUUGAUGAUAUAGUGAAAGGGUGUUUAGGUGCGUUGGAUACUGCGGAGAAGA
GUACUGGUAGUGGUGGGAAGAAGAGAGGUCCUGCUAUGUUUAGGA
.((((((((((..((((((((((((((....((((((.....(((.((.......)).)))...(((((.......
.((((((((.(.(.(.(((((((((.(((((......)))))...)))))...)))).).).))))))))))))))).))
)))).))....))))..))))))))..)))...))))))))).)).

CGGACAUGGCUUACUUCUUCUU
****
****

rsa-mir22
NAU_YH_CK-m0080 gi|161537956|gb|EY910368.1|EY910368:689:798:- 110(nt)
-57.70(kcal/mol)
CUGAUUCGCUUGGUGCAGGUCGGGAACUGAUUGGCUGACACAGCCACGUGGGUUUUCAUGGUUGGCUCGUGAGCAGGGA
UCGGAUCCCGCCUUGUAUCAAGUGAAUCCGA NAU_YH_CK-m0080 15
(.((((((((((((((((.(((((.((((((..(((.((((((((((((((....)))))..))))).)))..))).))
))))).)))))).)))))))))))))))).).
**********UGGUGCAGGUCGGGAACUGAU***
*CGGAUCCCGCCUUGUAUCAAG*********

rsa-mir23
NAU_YH_NaCl-m0042 Unigene6132_All:58:278:+ 221(nt) -98.70(kcal/mol)
UUCUAUUUUUAAUCCAUCUCCACAACCACCACUCUUCUCAUGCGAUCUCCGCUGCUCCGAGCCACGAAUCGGAGCCUUU
AUCUAUCGUUGUUAAGAAGGGCGGCGGAGGCGGCAGAGGCGGUGGCGGUUUCGGUGGAGGUGGUCGCAGUUUCGGUGGA
GGUGGUCGUAGUUUCGGUGGAGGCGGCAGAGGCGGUGGUGGAGGUGGAGGCGGAAAAGGUGGA
.((((((((..(((.(((((((..(((((((.(.((((.(((..((((((((((((((........))))))(((((
..............)))))(((((.(...(.((.(((((.(((((.(((((....))))).))))).))))).)).).
..).)))))).....)))))))))..))))))).))))))))..))))))).))))))))))))
****
****
********************************CGGUGGUGGAGGUGGAGGCGG**********

rsa-mir24
NAU_YH_NaCl-m0006 CL10961.Contig1_All:645:822:+ 178(nt) -41.20(kcal/mol)
ACUCGCAACUUUAGUCGGUUAGCUUGGAAGCCAAAAAGGUCGAUUGUAUGCUUUAAAGUGCGAGUAGUGGGAUAAUUAA
CUUAAAAUCUCGGAUUUAAUAGCUUAUUAGUAGAAGGUGGAUGCGUUACUUUGAAUCGAUUGAAGCUUUUGUUUUCUGA
GAAAAUGGGCAGAGAAGAGA NAU_YH_NaCl-m0006 11
.(((....((((.(((.(((..((((((((.(((((...(((((((((.(((...((((.((((...((((.......
))))....)))).))))...))).))...((..(((((((.....)))))))..)))))))))...))))).)))))))
)..))).)))))))..))).
***************CGGUUAGCUUGGAAGCCAAAA**
**UUGUUUCUGA
GAAAAUGGGC**********

rsa-mir25
NAU_YH_CK-m0038 Unigene31262_All:3:108:- 106(nt) -50.60(kcal/mol)
GAUUUGCUGUCAUGCCUGGCUCCCUGUAUGCCACAAGUGGAUAUCGAUUUUGGCUGAAAAAUUAGCUGCUGGUGGCGUA
CGAGGAGCCAAGCAUGACCAGAGGAAG
......((((((((.((((((.(((((((((.(((((.....((((((......))))))..))))).))))))))
)).))))))).)))))).)))......

**CGUA
CGAGGAGCCAAGCAUGA**********

rsa-mir26
NAU_YH_CK-m0011 CL2448.Contig4_All:345:478:+ 134(nt) -21.44(kcal/mol)
GAUUCUCUGUCAUAUAUUUGAAGGAUUGUUUGAGAGUUUCUGCAGGGGGUGAAGAAGAAGAAGAAGAAGAUGUCGAUGG
AAAAAAAAUUCAGAUCACGUUUCCUAUGGAAACUGUUAACGGCGACGGUAUAUU
.....(((.(((......))).)))((((((((.((((((((.(((((((((.(((.........................
.........)))...))))..))))).)))))))))).))))..)))).........
****
***********************CUAUGGAAACUGUUAACGGCG**********

rsa-mir27
NAU_YH_NaCl-m0057 gi|154259137|gb|EV570158.1|EV570158:1:113:+ 113(nt)
-42.30(kcal/mol)
GCGUCGCGAAUCCGCACGGUCAAGGCGCUACGUCGUCGUAUCCGACGAGCAGUUUGAAUAUGCGGAGAUCUGCGAUGAC
GAACUCGCUUGUGAGGAUCGGUGGCGUUGAAGGA
(((((..((..((....))))..))))).((((((((.((((.((((((((((((....(((((....)))))....)
))))).)))))))..))))))))))))).......
****
CUCGCUUGUGAGGAUCGGUGG*******

rsa-mir28
NAU_YH_NaCl-m0043 Unigene9448_All:109:184:- 76(nt) -18.00(kcal/mol)
AGCUCCUGCGAGAGCACAGGAGAAUUGUAAAUAGUUUUUUUUUUACUGUUUAGGGUUGUUUGUUUUUGUUUUUUUC
.......((((((((...((.((((.((((((((.........)))))))).)))).)))))))))).......
CUGUUUAGGGUUGUUUGUUUU*******

rsa-mir29a
NAU_YH_CK-m0114 gi|167492988|gb|FD977184.1|FD977184:116:432:- 317(nt)
-68.40(kcal/mol)
GGUAAUAUGGCUUGCAUAUCUCAGGAGCUUUAACUUACCCUUUAAUUGCUUUUACCCUUCUUUGGAUUGAAGGGAGCUC
UACAUCUUCUUUCACUUUAUCUAUAUCUAUUUCUCUAUAGUAAUUAAUGAAUUCAGUGUUGAUUUUGAUUACUUUUGCA
GUGACAAGCGCAUUCGUCUUGAGGAGUAUUUUUUGUGGUGAUGGUUUUGACUUUUGAUCCUUAAGUAAUGUAUGCUGUG
GUGUUUAAUUGAUUUUCUUGCUUGUGUGUAACUAGGGUUUAUGUAAAUGCUUCGGUAAUAUGCGAAGAUCCAUAUUUAC
A
(..((((((((((((((..(.(((((((((....(((((............((((((........))))))(((.(
((((....((((((((.((..................(((((((((.....(((....)))..)))))))))..)).)
)))).))).((((.((((((((((((..........(((.(......).))).....))))))))..))).))))))))
).)))............((((......))))..))))).....)))).))))).)..))))))).)))..)))))))..)
.
**********CUUGCAUAUCUCAGGAGCUUU***
****
****

****
*

rsa-mir29b
NAU_YH_CK-m0122 gi|167498764|gb|FD981419.1|FD981419:384:699:+ 316(nt)
-67.80(kcal/mol)
GGUAAUAUGGCUUGCAUAUCUCAGGAGCUUUAACUUACCCUUUAAUUGCUUUUACCCUUCUUUGGAUUGAAGGGAGCUC
UACAUCUUCUUUCACUUUAUCUAUAUCUAUUUCUCUAUAGUAAUUAAUGAAUUCAGUGUUGAUUUUGAUUACUUUUGCA
GUGACAAGCGCAUUCGUCUUGAGGAGUAUUUUUUGUGGUGAUGGUUUUGACUUUUGAUCCUUAAGUAAUGUAUGCUGUG
GUGUUUAAUUGAUUUUCUUGCUUGUGUGUAACUAGGGUUUAUGUAAAUGCUUCGGUAUAUGCGAAGAUCCAUAUUUACA
(..((((((((((((((.((.(((((((((....(((((.............((((((........))))))(((.(
((((....((((((((.((..................(((((((((.....(((....)))..)))))))))..)).)
)))).))).((((.(((((((((((...........(((.(......).))).....))))))))..))).))))))))
).)))............((((......))))..)))))).....)))).))))))).)))))).)))..)))))))..).
**********CUUGCAUAUCUCAGGAGCUUU**
****
****
****

rsa-mir30
NAU_YH_NaCl-m0041 Unigene4148_All:167:401:- 235(nt) -76.30(kcal/mol)
CAUAAGAGCUCUCUCUUGGUCCGCUUAGGCCAUAGAAAGCAGCAGCAUCAACAUCAACAACGUUUACCCAUUUGUUUAG
UCACUAGAGGACCACAAAGAACCAUAGUUAUAGCCCUCCUACCACUAUAUCUCAUCUUGAGCAUGUCCAACCCACACAC
ACACAUUGAACGAUUGUAAACGUUGUUACUGUUGCUACCGCCAAUGGACUUAGAUGGGCCAAGAGAGAGCUCAAGAU
.....(((((((((((((((((.(((((((((.(..((((((((.........((((((((((((....((((((((
(.....((((.........................))))........((((((((...)))).)))).............
....))))))))))..))))))))))))).)))))))))).....).)))).))))).)))))))))))))))))))).....
****
****
GACUUAGAUGGGCCAAGAGAG********

rsa-mir31
NAU_YH_CK-m0029 Unigene14582_All:1:115:+ 115(nt) -27.20(kcal/mol)
AUGGAGACUGGUCAGGGAGGAGGAGGAGGAGUAACUGAUUACGGAGGAGGAGGAGGUGGACUUUACGACGGAGCGAUGA
ACCCAUUCAAUCCAGGGUUUUAUGACCAACCUCCAA
.(((((..((((((.((((..((((((((.((.........((..(..((((........)))).)..))..........
)))).)))..)))....)))).)))))))..))))).
***GAGACUGGUCAGGGAGGAGGA**
****

rsa-mir32
NAU_YH_NaCl-m0037 Unigene3614_All:894:1040:- 147(nt) -58.30(kcal/mol)
UAGAGAGAGAGAGCAGACAGUAGUGUGAGAAAGUAGAUCUGGUUGGGUCCGGCGUACGGCCGUCGCGUGAGCGAGCAUG
CCGUCGCCGGAACCUUUUUUAUUCCGGUUAAAAUGUCCUUCUCUUACUUCUCUUUCGUCUCCUCUCAC

..(((((.((((..(((.((.((((.(((((.(((...((((..((((((((((.(((((..((((....))))....)
)))))))))).))))........))))......)).).))))).)))).)).)))..)))))))))..
**********GAGCAGACAGUAGUGUGAGAA**
****

rsa-mir33
NAU_YH_CK-m0037 Unigene30642_All:84:322:- 239(nt) -35.40(kcal/mol)
AGGUAUCAUUUUUUGUUUGUGUUAGUAUCAGUCUGGUUAUUAGUUAUUUUCUCUCAUGGUUUCUUUUUUUUCUUUGCCA
UCAUGGUUUUAUCUAUAAACAAUUCGAAGCUCCCUUUGUUUCACAACAAUUGAUCUUCAAAUAACCAACACUUAAUAUA
UCUUUGUGUUAUUAUCGUUGUUUAACUGUUUUGUAAAUGUCGAUAUUAGUGAUGGGCCUAACUCACACCCAAAAGUUAG
CU
.(((.....((((((...((((.(((...((.((.(((((((((..........(((((..............))))
)..(((((............(((.((((.((...(((((....)))))..)).)))).)))))))).....(((((((
....)))))))..((((..(((((.(......))))))..))))))))))))).)).)).))).)))).)))))))...)
))
****
****
GAUGGGCCUAACUCACACCCA******
**

rsa-mir34
NAU_YH_CK-m0097 gi|166139020|gb|FD570951.1|FD570951:1:137:+ 137(nt) -38.30(kcal/mol)
UUUUUCCACAGCUUUCUUGAACUUCCUUUUUCAUUUCCCUUCUUUUAAAGAUGUAAUAACUAAAGAUUGUUUUUAAACAUUUA
ACACUCUAGAAACAAAAAAAAAAGCUCAAGAAAGCUGUGGGAAAACAUGUCAGG
(((((((((((((((((((.(((..(((((..((((.......(((((....(((.(((........))).)))....))))
).......))))..)))))..))).))))))))))))))))))).........
****
**********************GCUCAAGAAAGCUGUGGGAAA**********

rsa-mir35a
NAU_YH_CK-m0016 CL7248.Contig1_All:113:261:- 149(nt) -61.40(kcal/mol)
UCUUUUGAGGGGAAUGUUGUCUGGCUCGAGGACUCUGGCUCUAUCUACUCAUGUUGGAUCGUCUUCGAUCUAAUCGAAUCAAA
CUUCUAUGUUUGAGAUCUGAUUAGGGUUUUAGCGUCGUCGGACCAGGCUUCAUUCCCCCCAAUUAU
....(((.((((((((..((((((.((((.(((.(((((((((((...((..(((((((((....))))))))).....(((((
(......))))))))...)).))))))..))).))).)))).)))))))..)))))))).)))....
**********GGAAUGUUGUCUGGCUCGAGG**
****

rsa-mir35b
NAU_YH_NaCl-m0026 CL7248.Contig2_All:66:137:- 72(nt) -20.00(kcal/mol)
GACAAGGAUUCUUCGACGGGAAGGGGCUUUCUCUUUGAGGGGAAUGUUGUUUGGCUCGAAGACUCUAGCUA
NAU_YH_NaCl-m0026 65
.....(((.(((((((....(((.(((.(((((((...))))))).))).)))...))))))).))).....
************UCGACGGGAAGGGGCUUUCUCU**************************************

GGAAUGUUGUUUGGCUCGAAG*******

rsa-mir36
NAU_YH_CK-m0030 Unigene1698_All:2193:2287:+ 95(nt) -24.90(kcal/mol)
GCGGAGACAUGGAGGAGGAGGAAGAAGAAGAGGAAAAGCUUCAGUGACAUUUUUAUUUUACUUCUUUUUUUUUUGGCAAAGCU
CUAAAGUCCGCU
((((....(((((..(..((((((((((((((((.................)))).))))))))))))))..)....))
)))...))))).
**********GGAGGAGGAGGAAGAAGAAGAG***
****

rsa-mir37
NAU_YH_CK-m0025 Unigene10071_All:60:178:+ 119(nt) -53.10(kcal/mol)
UAGAGUAUAAUGCAGCCAAGGAUGACUUGCCGGAGCGUUAUUAAUCAUGCGUAAAAAAAUAUUGAUGAUAAUGAUGAACAUAA
UUCUGGCAAGUUGUCCUUCGGCUACAUUUUGCUCUC
.(((((.((((.(((((((((((((((((((((((((((.((((..(((......)))..))))))))))))).......
))))))))))))))))).)))).)))).)))))).
****
****GGCAAGUUGUCCUUCGGCUACA**********

rsa-mir38
NAU_YH_CK-m0035 Unigene28808_All:80:231:- 152(nt) -20.40(kcal/mol)
AUUAGAAUUUAGGGUUUAGUGUUCUAUAUUAUUUAGAAAUAUAUUUUGAUAUUUGAAUUUAGGAUUUACAUUUAGAUUAAUAU
UUACAUUUGGAUUAGGGUUUAGAAUUGGAUUAGGGUUUAGUGAUUAGAAUUUAGGGUUUAAUAUUUAUA
..((((..(((((.(((((.((((((.((((((.(((....((.((((((.(((((((((..((((((.((.(((((....))
))).)).)))))).))))))))))))))).))...))))))))))))))))))).))))))..))))..
****
*************************************UAGUGAUUAGAAUUUAGGGUUU**********

rsa-mir39
NAU_YH_NaCl-m0064 gi|158647103|gb|EX756994.1|EX756994:1:139:+ 139(nt)
-22.80(kcal/mol)
GGGAGAUCUGAGUUCACUCUUUACGUCGAUUAGGGGAUUUUACGUUUUACUCGUCCUUUUUGAUUUAUUUUUUGUUUUGUUGA
AUGCCCAAUGAUGGAUAAAUUUUUAUAUAGAGCGGUUGUAUUUGGAUAACACUCCG
.(((((((((((.(((((((((.(((.(((((((.((((.(((....((.......................))..))).))
)).)))..)))).)))..........))))).)).)).))))))))....)))).
****
*************************UAUAGAGCGGUUGUAUUUGGA**********

rsa-mir40
NAU_YH_CK-m0126 gi|332773864|gb|FY434434.1|FY434434:154:265:+ 112(nt)
-33.20(kcal/mol)
AGAGGGUAGAAAUAUCUGCCGACUCAUCCAUCCAAACACUCAUGGUUAUGAAACAAGAAAUGUUAAACCACAGUGACUGUGUA
UGAAUGAUGCGGGAGAUGUUUUUCAUCUC

.((((..((((((((((.(((..((((.((((((..((((..(((((....((((.....)))).))))).))))..)).).)
)).)))))..))).))))))))))..))))
***UA
UGAAUGAUGCGGGAGAUGU**********

rsa-mir41
NAU_YH_NaCl-m0038 Unigene36179_All:834:1030:- 197(nt) -37.70(kcal/mol)
CUCAGCCGAGGUUCCAUUACCACGCUUUCAGAUUUUAUUCCUAAGGUUACAAAAGUUUUUGUUAAUACAAAGGUUGAUAAAC
UCAUUAUAUAGAGAGUACUCUGUUUCUUAUAACAAAGUUUGAUAAACACAAGAGUAGCUAAUGCAAGUACUCUUCUUCUUUUA
CUCCGUCCUGGUCUUGGCUUUCUUGGUAGAU
.((.(((((((..(((..((((.(((((..(((((((....)))))))...))))).((((((....))))))(((..(((((
(..(((((.(((((........))))))))))...)))))))...)))..(((((((((....))...)))))))).........
........))))..)))...))))))).)).
*UCAGCCGAGGUUCCAUUACCAC**
****
****

rsa-mir42a
NAU_YH_NaCl-m0081 gi|166105866|gb|FD536894.1|FD536894:404:575:- 172(nt)
-46.20(kcal/mol)
UAGCCUUUGCGGGGGACACGGAUAAAGGAAUGUUGGAUGUGUUGAACGGAUGCAAGAAGCUGAGGAAGCUGGAGAUUAGGGAU
AGUCCGUUUGGGAACGCUGCGCUUCUUGCUGAUGUCGGUAAGUACGAAACAAUGCGGUCCCUUUGGAUGUCGUCUUGUGAAGU
AACGCU
.((((((((((((((((....((((((.((((((..((((((..((.((((((((((((..((....((.(((((((....)
))))...)).))....))..))))))))))....))).)).))))))...))))...)).)))))))..)))).))))))))))).
...)))
****
UCCCUUUGGAUGUCGUCUUGUG*
******

rsa-mir42b
NAU_YH_NaCl-m0092 gi|166143240|gb|FD575456.1|FD575456:357:491:- 135(nt)
-31.20(kcal/mol)
CGUGUUGAAUGGGUGUAAGAAGAUGAGGAAGCUGGAGAUAAGAGACAGUCCGUUUGGGAACGCUGCGCUUCUUGCUGAUGUUG
AUAAGUACAGAACAAUGCGAUCCCUUUGGAUGUCGUCUUGUGAAGUAACGCU
(((.....)))(((((..............(((....((((((((((.((((...((((.(((...(.((((((((.((....
)).))))).))))))...))).))))..))))))))).)))))))..))).))))))
****
********************UCCCUUUGGAUGUCGUCUUGUG**********

rsa-mir43
NAU_YH_NaCl-m0052 gi|154178536|gb|EV547876.1|EV547876:570:715:+ 146(nt)
-44.20(kcal/mol)

UCGUGUUAGUUCUGGAUUUGGAGUGGUUUUUAUCUAGCUACCGGCUGUGUGAAUAAGUAGUGUUCGGGAAGCUUUUAGAGGCU
UCGCCGAGAGGUGUUGGUGCGUAUGAGGCGCGGGGAAAGCCUACUCAAAGUCCAAUGGCGAGU
.(.((((....((((((.((((((((((..(((((.(((.(((((.......))))).((((((((((....)))))
)).))))).))))))))((((.....)))))..))))))).)))).))))))).))))).).
**********UCUGGAUUUGGAGUGGUUUUUA***
****

rsa-mir44
NAU_YH_NaCl-m0013 CL2699.Contig1_All:931:1177:+ 247(nt) -57.00(kcal/mol)
UGGACUCCGAUGAAGUGAGGAUGAUAGGGAUUUGGGGUCCAUCUGGAAUUGGUAAGAGCACCAUCGCAAGAUUUCUAUUUAGC
CAACACUCUGGUGACUUCCAACUUAGCGUCUAUAUGGAGAAUAUCAAAAGAGAGUAUCCAAGACCUUGUUUCGAUAGAUACAG
CGCACAACUGCAAUUACAGAAUAAGUUCUUGUCUCUAAUACUCAAUCAGAAGGAUAUCACUAUCCAUCAUUUAGGAGUUGC
..((((((((..((((.(((.(((((((((((((((((...(((((.(((((.(((.....(((....))).....))).))
)))...))))).))))))......((.((((...((((.....((....)).....))))))))))(((.((....)).))).
.......(((......)))...))))))))))))))..))))..))).((.(((((....))))).)).....))))))..
**********UGAAGUGAGGAUGAUAGGGAUU***
****
****

rsa-mir45
NAU_YH_NaCl-m0112 gi|332783936|gb|FY434072.1|FY434072:518:628:- 111(nt)
-50.10(kcal/mol)
UAAUAUUUUUGAGCGGCAACUAUUGUAGGUUUCAAACUAUAUUUGACCCUAUAUUAAUACUGAUCCUUAUAGUUUGAAAUCU
ACAAUAGUUGCAGCUGAGAAGAUAUUAU
((((((((((.(((.((((((((((((((((((((((((........................)))))))))))))))
))))))))))).))).))))))))))).
**********UGAGCGGCAACUAUUGUAGGU**
****

rsa-mir46
NAU_YH_CK-m0100 gi|166146391|gb|FD580445.1|FD580445:1:106:+ 106(nt) -42.10(kcal/mol)
GGAAGAUCUCUAUUGGCAGGAAACCAUUACUUAGAUCUUUGCAUCUCUCUGUGCAUUGAUUUGUAGUGAGUUCUCUGCCAAAG
GAGAUUUGCCCGGUAAUUCUUCU
((.((((((((.((((((((.(((((((((..(((((..(((((......)))))..))))))))))).))).)))))))).)
)))))))..))............
***UGCCAAAG
GAGAUUUGCCCGG**********

rsa-mir47a
NAU_YH_NaCl-m0080 gi|166102495|gb|FD536486.1|FD536486:2:129:+ 128(nt)
-43.10(kcal/mol)
GGAAAGUCACUGGAGGCAGCGGUUCAUCGAUCUCUUCCUGUGGAUUUUUUGUUUGUUUCUUUGUUGUUGUUGGAAAGAGAUU
AAAAGCACGAACAGAUCGAUAAACCUCUGCAUCCAGCGGUUGCCU

```
((..((((.(((((.((((.((((.((((((((.(((.(((.....((((.((((.(((((...........))))).)))).
))))))).))).)))))))).)))).)))).))))).)))).)).
**********UGGAGGCAGCGGUUCAUCGAUC***************************************************
*********************************************

rsa-mir47b
NAU_YH_NaCl-m0096        gi|167442794|gb|FD947716.1|FD947716:275:406:+        132(nt)
-42.00(kcal/mol)
UGACAGUUGCUGGAGGCAGCGGUUCAUCGAUCUCUUCCUGAGAAUUUUUUUUUUGGAAAGAAAGGAAGAUAAAAAUUAAAAGC
AUGAACAGAUCGAUAAACCUCUGCAUCCAGCGGUUUACCUCUUCGUCAA
((((((((((((((.((((.((((.((((((((.(((.((..(((((((.((((...........)))).))))))).....)
).))).)))))))).)))).)))).))))))))))).........)))).
**********UGGAGGCAGCGGUUCAUCGAUC***************************************************
*************************************************

rsa-mir47c
NAU_YH_NaCl-m0032 Unigene13287_All:70:200:+ 131(nt) -41.80(kcal/mol)
UGACAGUUGCUGGAGGCAGCGGUUCAUCGAUCUCUUCCUGAGAAUUUUUUUUUGGAAAGAAAGGAAGAUAAAAAUUAAAAGCA
UGAACAGAUCGAUAAACCUCUGCAUCCAGCGGUUUACCUCUUCGUCAA
((((((((((((((.((((.((((.((((((((((((((.....(((((....)))))...)))))))...............
......))))))).)))).)))).))))))))))).........)))).
**********UGGAGGCAGCGGUUCAUCGAUC***************************************************
************************************************

rsa-mir47d
NAU_YH_NaCl-m0044 Unigene9985_All:220:349:- 130(nt) -45.60(kcal/mol)
UGACAGUUGCUGGAGGCAGCGGUUCAUCGAUCAAUUCCUGUGAAUAGUGAUGUUGGAAACAAUUUUACUUGGAAAGCAUGAAU
CGAUCGAUAAACCUCUGCAUCCAGUGCUUCUCCUUCCUGAUUUGUCU
.(((((..((((((.((((.((((.(((((((.((((.(((....(((((.((((....)))).)))))......))).))))
.))))))).)))).)))).))))))................))))).
**********UGGAGGCAGCGGUUCAUCGAUC***************************************************
***********************************************

rsa-mir48
NAU_YH_CK-m0027 Unigene11487_All:25:204:- 180(nt) -70.60(kcal/mol)
CUUACUGUGGGAGAAAACGCCUUUACAUUCACAGUAGGUUAGUGGUGCGCUUGUUGUUACUCGAAUCCAAAACUUCACAUUUU
AACAAUUCUCCCACAUGACAAGCUGUCACAAGAUCAAUAACGACAACCGCACCAAUAACCUAUCGGUGGAUGUAGAGGCAUUU
CUUCCCACGAUAAA
.(((.(((((((((((..((((((((((((((.((((((((.(((((((.(((((((((...(((........))).......
...............(((((...)))))........))))))))).))))))).))))))))..)))))))))))))).)))
).))))))).))).
***********************************************************************************
******************************************************************UGGAUGUAGAGGCAUUU
CUUC**********
```

rsa-mir49

NAU_YH_NaCl-m0048 gi|154171319|gb|EV542593.1|EV542593:137:238:- 102(nt)
-19.30(kcal/mol)

CUGGUCCCUUAUAAAUAAUAUAGAUUCUAGAUAUUUUUAAUUACUAAUUUAUCUUAUUACUUGGUGAAGGUGGUUGGUAAUUA
GAUUAGGUGUUUGAGACCG

.(((((..(((((.....)))))....((((((((((((((((((((((.((((..((((...))))))))))))))))))))
))...)))))))).)))))

**UGGUUGGUAAUUA
GAUUAGGUG**********

rsa-mir50

NAU_YH_CK-m0009 CL1950.Contig3_All:82:181:- 100(nt) -29.10(kcal/mol)

AGCGUGAGAGUGUAUGUUACAGAUGUGGAUGUAAGGGACAUUGGUCUCGUACCUGUCGUACUCCUCCACAUCUCUGUAAGUUA
UAUCAAGAGUCCACGAA

..((((.(((((((..(((((((((((((.(...(.((((..(((.....))))))).)...).))))))..)))))))..))
)))......))))))..

**********UGUAUGUUACAGAUGUGGAUG**
****

rsa-mir51

NAU_YH_CK-m0043 gi|154092983|gb|EV525956.1|EV525956:339:419:+ 81(nt)
-30.70(kcal/mol)

UUGGCGGGAAAUUGCGUUUUUCCAGUUUUGACGGGAAAUUGCGUUUUCUGGUUUUGACGGGAAAUUGCAUUUUCCGGUU
UU

..(((.(((((.((((.((((((.(((..(((.(((((......))))).)))..))))))))).)))).))))).)))
..

UUUGACGGGAAAUUGCAUU******
**
